# The ion transport, GPCR, and RTK toolkit expression in the human cerebrovascular endothelial cell line, hCMEC/D3: an Omics perspective

**DOI:** 10.3389/fphys.2025.1733266

**Published:** 2025-12-18

**Authors:** Giorgia Scarpellino, Valentina Brunetti, Francesca Scolari, Luca Visentin, Gerardo Rosario Biella, Federico Alessandro Ruffinatti, Francesco Moccia

**Affiliations:** 1 Laboratory of General Physiology, Department of Biology and Biotechnology “L. Spallanzani”, University of Pavia, Pavia, Italy; 2 Institute of Molecular Genetics IGM-CNR, Pavia, Italy; 3 Department of Life Sciences and Systems Biology, University of Turin, Turin, Italy; 4 Laboratory of Cellular and Molecular Physiology, Department of Medicine and Health Sciences “V. Tiberio”, University of Molise, Campobasso, Italy

**Keywords:** hCMEC/D3, blood-brain barrier, RNA-Seq, transporters, pumps, ion channels, G-protein coupled receptors, receptor tyrosine kinases

## Abstract

The blood-brain barrier (BBB) plays a central role in maintaining the ionic milieu required for neuronal activity and in translating neuronal activity in a local elevation in cerebral blood flow (CBF). However, the molecular repertoire of the human BBB remains poorly defined. Here, we performed a systematic transcriptomic analysis of 672 genes using eight independent RNA-Seq datasets generated from the human brain endothelial cell line hCMEC/D3, the most widely used *in vitro* model of the human BBB. We focused on ion channels, ion transporters, G protein–coupled receptors (GPCRs), and Receptor Tyrosine Kinases (RTKs), which govern ionic homeostasis, barrier integrity, and CBF. Among the most abundantly expressed ion transporters were subunits of the mitochondrial F-type ATPase complex (F-type ATPase α subunit, F-type ATPase β subunit, F-type ATPase C subunit), reflecting the high metabolic demands of the BBB. Key regulators of intracellular Ca^2+^ homeostasis, including SERCA2, PMCA1/4, and SPCA1, were consistently detected, supporting efficient Ca^2+^ clearance across endoplasmic reticulum (ER), plasma membrane, and Golgi compartments. Our analysis of ion channels revealed a selective repertoire with prominent expression of Cl^−^-permeable channels (CLIC1/4, CLNS1A, VDAC1-3, VRAC) and various K^+^-permeable channels, including IK_Ca_/K_Ca_3.1, K_IR_2.1, K_Na_1.2, BK_Ca_, K_V_4.1, and TREK-1. Na^+^-permeable channels (ENaC and NALCN), non-selective cation channels (TRP, HCN2/3), and ER- (InsP_3_Rs, TRICs, and putative leak channels), and lysosomes-associated (TRPML1 and TPCs) channels were also detected. Additionally, we identified transcripts for mechanosensitive channels (PIEZO1, TACAN, TMC7, TMEM63B) and gap junction proteins (Cx43, Cx45, Cx47), as well as a broad array of ionotropic and metabotropic receptors, including purinergic, adenosine, histamine, GABA, adrenergic and nicotinic receptors. Growth factor-related RTKs (FGFR, IGFR, EGFR, PDGFR, VEGFR) were consistently expressed, underscoring their role in angiogenesis, endothelial-pericyte interactions, and BBB integrity. This meta-analysis highlights the conserved expression of transporter genes across datasets, contrasted with lower and more variable expression of ion channels and receptors, suggesting that the latter may be context-dependent and dynamically regulated. These findings provide a reference framework for understanding the human BBB transportome, offering new insights into the molecular toolkit of the human BBB to support future investigations into the role of endothelial ion transport in neurological disorders.

## 1 Introduction

The blood-brain barrier (BBB) is a highly specialized physical barrier between the blood and the interstitial brain fluid (ISF), playing an important role in maintaining a precisely regulated microenvironment for reliable neuronal signalling (Kaplan et al., 2020) and in translating neuronal activity in a local elevation in cerebral blood flow (CBF), according to a mechanism known as neurovascular coupling (NVC) (Negri et al., 2021d; Moccia et al., 2022). The BBB is established by the association of capillary endothelial cells (cECs), pericytes, astrocytes, and neurons, that together constitute the neurovascular unit’ (NVU) (Kaplan et al., 2020). The NVU is critical for inducing and maintaining structural and functional integrity of the BBB by conferring specific features to cECs. Brain cECs lack fenestrae, show limited transcytosis, and are tightly interconnected by adherens and tight junctions. This complex organization restricts paracellular diffusion and shields the central nervous system (CNS) from blood-borne toxic compounds, thereby preserving the ionic and chemical composition of the neuronal milieu required for proper neural circuit function (Abbott et al., 2010; Kaplan et al., 2020; Benz and Liebner, 2022). Due to the high trans-endothelial resistance and the substantial lack of vesicular transport, the majority of solutes that must be exchanged between the blood and brain require a variety of mechanisms, including receptor-mediated transcytosis and active saturable transporters, such as solute carrier-mediated transport and active efflux pumps, as well as ion and water channels, ion pumps and exchangers (Abbott et al., 2010; Benz and Liebner, 2022). The movement of ions across the BBB is ensured by a rich array of ion channels and transporters for Na^+^, K^+^, Cl^−^, HCO_3_^-^, Ca^2+^ and other ions, asymmetrically distributed between the luminal and abluminal membranes of the BBB endothelium (Abbott et al., 2010). The most extensively described BBB transport mechanisms mediate the trans- or paracellular movement of hydrophilic solutes, e.g., sugars, amino and fatty acids, and xenobiotics, across brain capillaries, either into or out of the brain parenchyma, which could be targeted to improve drug delivery to the CNS (Pulgar, 2018; Rust et al., 2025). In contrast, despite their fundamental role in regulating the neural microenvironment, endothelial ion channels and transporters at the BBB are often overlooked (O'Donnell, 2014).

In addition to regulating the ionic microenvironment around central synapses and axons, the endothelial ion transport machinery is emerging as a crucial regulator of BBB function (Stoica et al., 2021; Tang et al., 2025) and NVC (Negri et al., 2021d; Moccia et al., 2022). Studies on mouse brain microvasculature showed that cECs express a variety of G-Protein Coupled Receptors (GPCRs) and ionotropic receptors as well as chemo- and mechanosensitive ion channels, which enable them to integrate neuronal (e.g., neuronal firing and synaptic activity) and blood-borne (e.g., circulating autacoids, pulsatile stretch and shear stress) signals, thereby modulating BBB permeability and cerebral blood flow (Kaplan et al., 2020; Alvarado et al., 2021; Negri et al., 2021d; Stoica et al., 2021; Moccia et al., 2022; Soda et al., 2023; Lim et al., 2024; Scarpellino et al., 2024; Tang et al., 2025). Endothelial membrane receptors primarily translate chemical and physical cues into changes in resting membrane potential (V_M_) and intracellular Ca^2+^ concentration ([Ca^2+^]_i_) (De Bock et al., 2013; Negri et al., 2021d; Stoica et al., 2021; Moccia et al., 2023b). It has been shown that mouse brain cECs may detect neuronal activity through the inward-rectifier K^+^ (K_ir_2.1) channel, which initiates and sustains the propagation of endothelial-dependent hyperpolarization (EDH) from capillaries to upstream arterioles, thereby relaxing precapillary sphincters and enhancing blood flow to downstream microvessels (Longden et al., 2017). In addition, the structural integrity and permeability of the BBB is finely modulated by synaptically released glutamate, which gates endothelial N-methyl-D-aspartate (NMDA) receptors located on the abluminal side of the capillary tube (Kim et al., 2022). Similarly, an increase in [Ca^2+^]_i_ may increase the BBB permeability through the rearrangement of both adherens and tight junctions (De Bock et al., 2013; Stoica et al., 2021) and induce vasodilation at the post arteriole-capillary transitional zone by activating the endothelial nitric oxide (NO) synthase (eNOS), which mediates the production of the vasoactive gasotransmitter, NO (Longden et al., 2021). In accord, somatosensory stimulation has been shown to activate G_q_PCRs in mouse cECs, thereby leading to intracellular Ca^2+^ oscillations, NO release, and CBF increase throughout first-to third-order capillaries (Longden et al., 2021).

Emerging evidence indicates that cerebral disorders, including small vessel diseases, neurodegenerative disorders, and traumatic brain injury, impair BBB integrity and CBF regulation, by dismantling the endothelial ion signalling machinery (Peters et al., 2021; Moccia et al., 2022; Polk et al., 2023; Soda et al., 2023; Mughal et al., 2024b; Soda et al., 2024b; Rust et al., 2025). Therefore, identifying the main players in ion transport and GPCR signaling at the BBB is of fundamental importance to elucidate the physiological role of cECs within the human brain microcirculation and to design alternative therapeutic strategies for neurological and neurodegenerative disorders (Moccia et al., 2022). In the present investigation, we exploited gene expression data sets from RNA-sequencing studies on the hCMEC/D3 cell line, the most widely employed *in vitro* model of human BBB (Weksler et al., 2013; Helms et al., 2016; Qi et al., 2023), stored in the publicly available database databases of NCBI Sequence Read Archive (SRA) and European Nucleotide Archive (ENA). We studied the transcriptional expression of a list of 672 genes coding for ion channels, transporters, membrane ATPases, GPCRs, receptor tyrosine kinases (RTKs) based upon their documented or potential involvement in the regulation of brain cerebrovascular endothelium. We exploited a tailor-made bioinformatic tool, thus highlighting a signature expression profile with a potential physiological significance. We focus our attention on the GPCRs, RTKs, ion channels and transporters that, based on studies of mouse microcirculation, are most likely to be involved in ion transport, regulation of BBB permeability, and adaptation of CBF to metabolic neuronal demands.

## 2 Materials and methods

### 2.1 Literature search and RNA-Seq data processing

A systematic literature search was carried out to identify publicly available RNA-Seq datasets of the human brain microvascular endothelial cell line, hCMEC/D3. The NCBI Sequence Read Archive (SRA; https://www.ncbi.nlm.nih.gov/sra) was queried using the single keyword “hCMEC D3”. Studies not encompassing the whole protein-coding transcriptome (e.g., miRNA-focused studies) were discarded. Out of a total of 12 retrieved datasets, 8 were deemed suitable for our purposes and retained for subsequent analysis (see ENA and GEO accession numbers in Table 1).

**TABLE 1:** List of hCMEC/D3 transcriptomic studies collected for meta-analysis.

ENA BioProject ID	Study alias (GEO/AE)	Sample size	Library layout	Median read length	Average sequence depth	Uniquely mapped reads	Platform	References
PRJNA307652	GSE76528	8	PE	2 × 51 bp	57.1 M	78.9%	Illumina HiSeq 2000	Kalari et al. (2016)
PRJNA575504	GSE138309	3	PE	2 × 78 bp	22.9 M	91.3%	Illumina NextSeq 550	Simon et al. (2021)
PRJNA578611	GSE139133	2	PE	2 × 150 bp	24.3 M	95.3%	Illumina NovaSeq 6000	Fan et al. (2021)
PRJNA777606	GSE187565	2	PE	2 × 150 bp	27.8 M	94.2%	Illumina NovaSeq 6000	Cantu Gutierrez et al. (2025)
PRJNA847413	GSE205739	4	PE	2 × 150 bp	23.3 M	60.2%	Illumina NovaSeq 6000	NA
PRJEB48614	E-MTAB-11129	3	PE	2 × 41 bp	23.1 M	85.9%	Illumina NextSeq 500	Gil et al. (2022)
PRJNA667281	--	3	PE	2 × 150 bp	22.1 M	96.2%	Illumina NovaSeq 6000	Liu et al. (2021)
PRJNA896725	--	5	PE	2 × 150 bp	25.6 M	94.1%	Illumina NovaSeq 6000	Wu et al. (2024)

RNA-Seq studies are listed along with their main attributes, from left to right: ENA BioProject ID, Study Alias ID (GEO Series ID or ArrayExpress ID), usable Sample Size (i.e., only untreated control samples were retained in each study), Library Layout (SE = Single-Ended or PE = Paired-End reads), Median Read Length (in bp = base pair units), Average Sequency Depth (as millions of total reads per sample), percentage of Uniquely Mapped Reads, sequencing Platform technology, and related literature Reference (when available).

To maximize comparability across datasets and minimize potential computational batch-effects due to pipeline heterogeneity, we opted to download the raw reads (in FASTQ format) for all selected studies directly from the European Nucleotide Archive (ENA; https://www.ebi.ac.uk/ena/browser) and reprocess them from scratch by using the same standardized computational pipeline. For this purpose, we developed a dedicated suite of scripts in Bash, Python, and R that combines original code with wrappers of established third-party software, thereby covering the entire process from raw reads to count tables. This suite—named x.FASTQ—is publicly available on GitHub (https://github.com/TCP-Lab/x.FASTQ) and fully documented both in the repository and in a preprint published on Preprint.org (Ruffinatti et al., 2025).

In particular, x.FASTQ was first used to retrieve sample metadata from ENA and then, based on this information, to download the FASTQ files of interest, retaining only untreated control samples and excluding all treated samples from each study. This selection resulted in 30 usable paired-end RNA-Seq runs overall (see details in Table 1). The reanalysis pipeline then proceeded as follows: adapters and low-quality bases were trimmed using BBDuk (https://archive.jgi.doe.gov/data-and-tools/software-tools/bbtools/), reads were aligned to the human reference genome (GRCh38 Ensembl Release 110 assembly) with STAR (https://github.com/alexdobin/STAR) (Dobin et al., 2013), while transcript quantification was performed with RSEM (https://github.com/deweylab/RSEM) (Li and Dewey, 2011). At each step, quality control analyses were conducted, including read quality assessment with FastQC (https://www.bioinformatics.babraham.ac.uk/projects/fastqc/), integrated reporting with MultiQC (https://seqera.io/multiqc), and Principal Component Analysis (PCA) for the detection of potential outlier samples.

For each study, the resulting expression tables were summarized and annotated at the gene level, using Transcript Per Million (TPM) metric as a *quasi-absolute* expression measure (in arbitrary units) allowing comparisons of expression levels within a sample (Li and Dewey, 2011; Wagner et al., 2012). The complete set of outputs—including RSEM quantification tables, final expression matrices in TPMs, quality control reports, x.FASTQ logs from each step of the pipeline, and curated sample metadata—has been deposited in Zenodo and is freely available at the following DOI: https://doi.org/10.5281/zenodo.12729454.

We applied a TPM ≥1 threshold to define expressed genes. This choice follows common practice to exclude transcripts close to sequencing noise and to focus downstream analyses on reliably detected genes; TPM = 1 corresponds approximately to one transcript per million and is often used as a conservative cutoff that balances sensitivity and specificity for bulk RNA-seq data. To ensure that this threshold was also appropriate for our specific datasets, we examined the empirical distribution of log_2_ (TPM +1) values in each study. In all cases, the distribution showed a clear multimodal structure. To model it more formally, we fitted a three-component Gaussian mixture model: one component centered near zero to accommodate null (or quasi-null) expression values, a second component representing low-abundance/background transcripts, and a third component corresponding to genuinely expressed genes. We then defined the decision boundary as the intersection between the second (background) and third (expressed) Gaussian components. Notably, across studies this inferred boundary consistently fell close to TPM ≈1, thus providing independent quantitative support for the chosen cutoff. Two representative examples (for GSE76528 and GSE138309 series) are reported in Supplementary Figure S1, showing the mixture fit and the estimated boundary. We also acknowledge that some low-abundance transcripts (e.g., ion channels) can be functionally important despite low TPMs; this point is discussed in detail in the Discussion section.

### 2.2 Absolute expression profiling of transportome

For the downstream analysis of gene expression, we developed a custom R-based workflow called *Endothelion* (https://github.com/TCP-Lab/Endothelion), specifically designed to profile transportome expression in the selected hCMEC/D3 studies. Additionally, an R package named *SeqLoader* (https://github.com/TCP-Lab/SeqLoader) was also developed to efficiently manage the hierarchy of RNA-Seq data we collected for our meta-analysis, which consisted of multiple studies (or *series*), each potentially comprising multiple samples (or *runs*).

A comprehensive list of Genes Of Interest (GOIs) relevant to inorganic ion transport and homeostasis was assembled by integrating gene annotations from multiple sources, including the HUGO Gene Nomenclature Committee (HGNC) database (https://www.genenames.org/), the IUPHAR-DB (https://www.guidetopharmacology.org/), and Gene Ontology (https://geneontology.org/). This resulted in a curated panel of 672 GOIs, which comprised:

- all known human ion channels (436 genes, i.e., the complete channelome, including possible auxiliary or modulatory subunits),
- the entire set of aquaporins (14 genes),
- all ATPase pumps (90 genes),
- solute carriers (SLCs) specific for inorganic solutes (81 genes),
- a set of 51 receptors (GPCRs and RTKs) with roles in inorganic ion dynamics.

The complete list of GOIs is provided in Supplementary Table S1.

**FIGURE 1:**
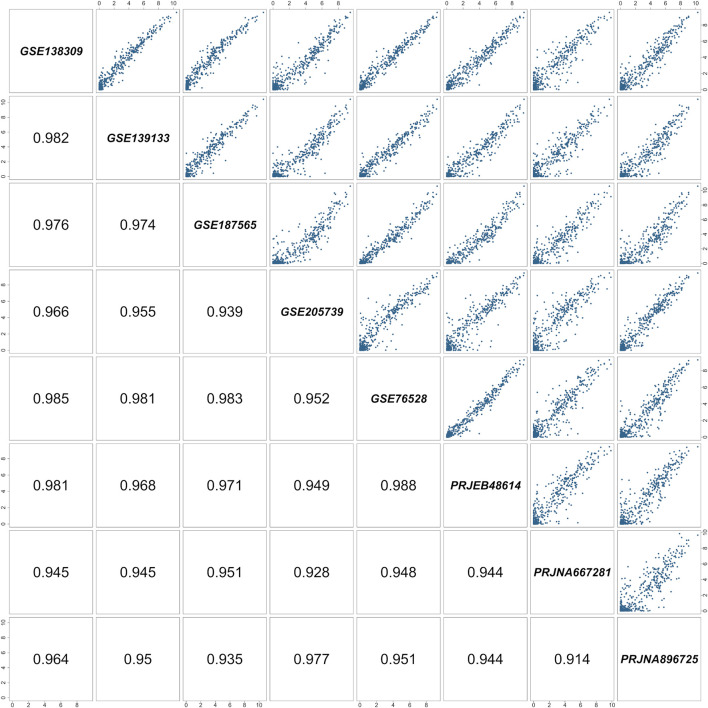
Pairwise correlation matrix of the RNA-Seq studies included in the meta-analysis. Each cell represents a comparison between two of the eight selected studies. Study identifiers are shown along the diagonal, pairwise scatterplots of quantile-normalized $\log_{2}\left(\text{TPM}+1\right)$ expression values of the Genes Of Interest (GOIs) occupy the upper triangle of the matrix, while the corresponding Pearson correlation coefficient values are reported in the lower triangle. All correlation values exceed 0.9, indicating strong concordance among datasets and limited batch effects.

Expression matrices generated through the x.FASTQ pipeline (see Section 2.1) were converted to log scale, as $\log_{2}\left(\text{TPM}+1\right)$ , merged into one global expression table, and then subjected to quantile normalization to correct for residual batch-effect across studies (see Figure 1). Expression data were subsequently filtered to retain only the genes present in the GOI panel and descriptive statistics were computed for each one of them, including the within-study mean, the overall (cross-study) mean, and the related 95% Confidence Interval (CI). In all cases, the number of independent observations (*n*) was equal to 8, corresponding to the number of studies included in the analysis.

**FIGURE 2:**
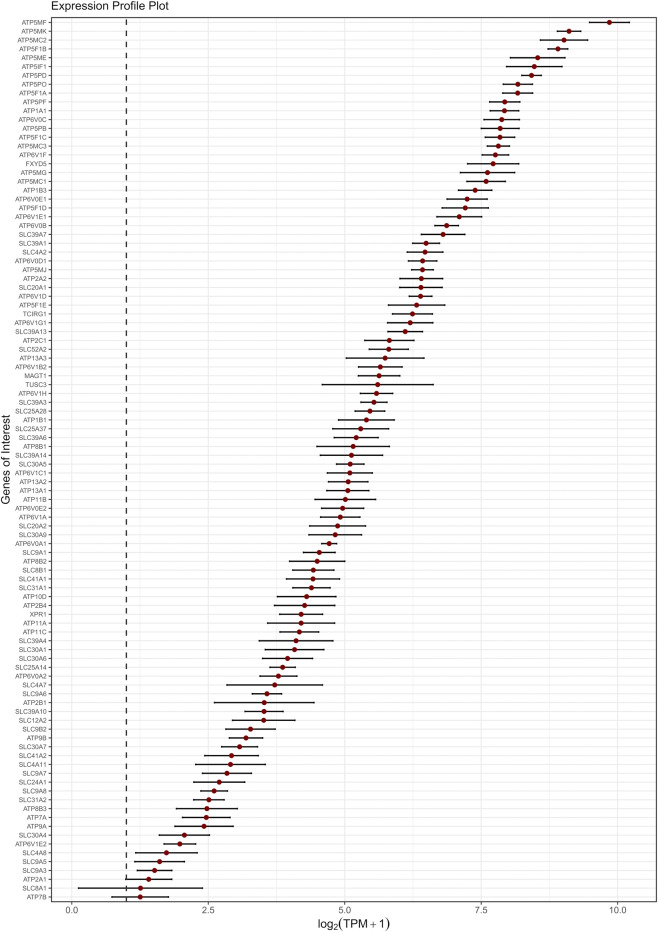
Expression levels of the top-expressed ion transporters (ATPase pumps and SLCs) in the GOI list. Genes are ordered along the y-axis by decreasing expression. The x-axis reports the mean $\log_{2}\left(\text{TPM}+1\right)$ values across studies, with each gene represented by a red dot and a horizontal bar indicating the 95% confidence interval (95% CI). The vertical dashed line marks the conventional expression threshold (TPM = 1).

**FIGURE 3:**
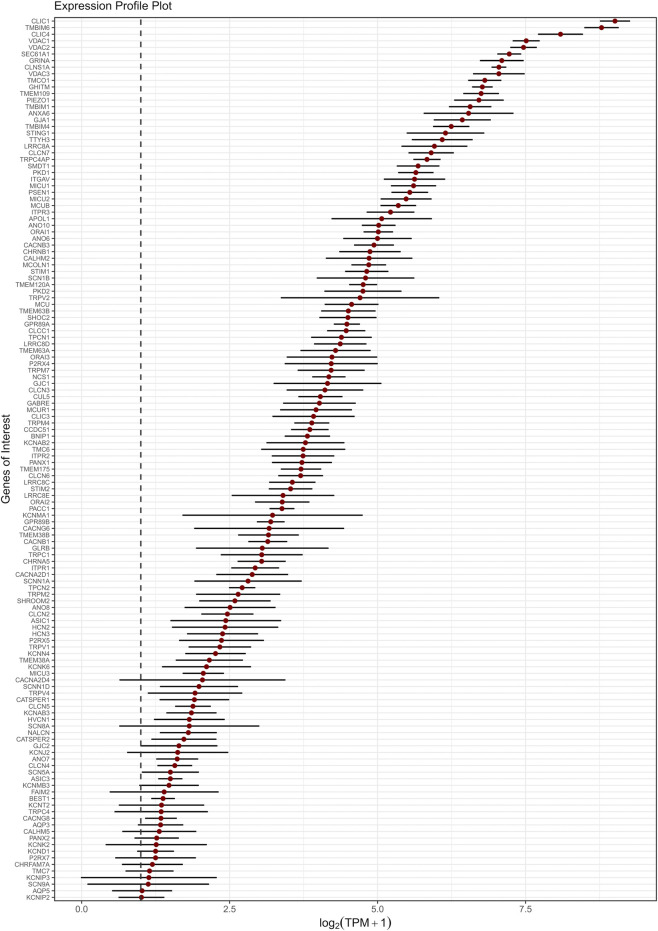
Expression levels of the top-expressed ion channels and aquaporins in the GOI list. Genes are ordered along the y-axis by decreasing expression. The x-axis reports the mean $\log_{2}\left(\text{TPM}+1\right)$ values across studies, with each gene represented by a red dot and a horizontal bar indicating the 95% confidence interval (95% CI). The vertical dashed line marks the conventional expression threshold (TPM = 1).

**FIGURE 4:**
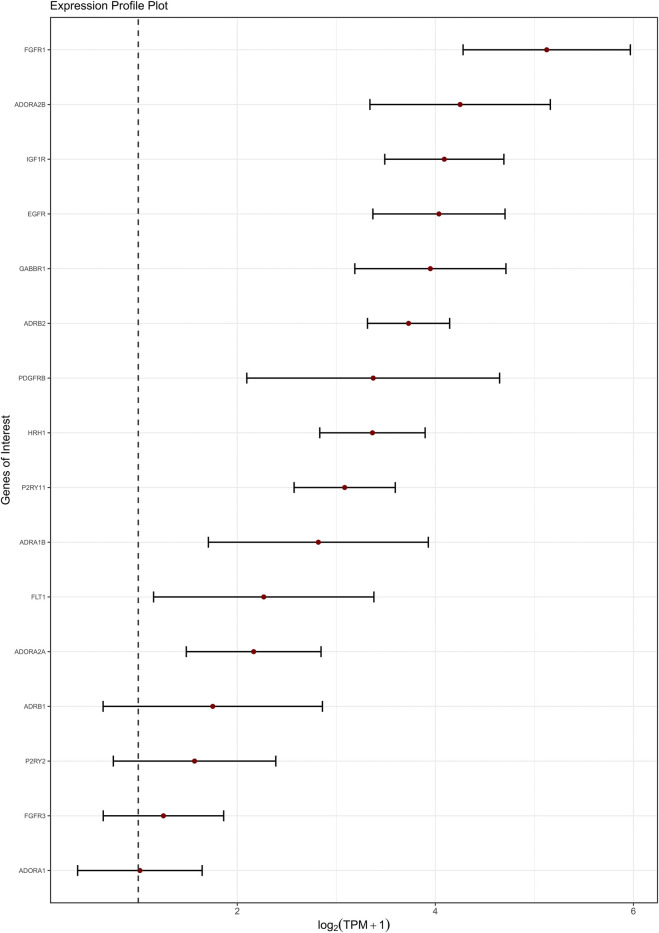
Expression levels of the top-expressed membrane receptors (GPCRs and RTKs) in the GOI list. Genes are ordered along the y-axis by decreasing expression. The x-axis reports the mean $\log_{2}\left(\text{TPM}+1\right)$ values across studies, with each gene represented by a red dot and a horizontal bar indicating the 95% confidence interval (95% CI). The vertical dashed line marks the conventional expression threshold (TPM = 1).

Finally, a conventional threshold of $\log_{2}\left(\text{TPM}+1\right)=1$ was adopted to classify a gene as expressed in hCMEC/D3 cells. GOIs that passed this filter were visualized as 95% CI bars, grouped by category into three classes: transporters (SLCs and ATPase pumps; Figure 2), pores (ion channels and aquaporins; Figure 3), and receptors (GPCRs and RTKs; Figure 4).

### 2.3 Data availability and pipeline reproducibility

All the software developed for the analyses described in this study—namely, x.FASTQ, Endothelion, and SeqLoader—is openly available through the GitHub organization page of the *Turin Cell Physiology Lab* (https://github.com/TCP-Lab).

In addition, to ensure full transparency and reproducibility, we implemented the entire analysis workflow within the *Kerblam!* project management open-source framework (https://www.kerblam.dev/) (Visentin et al., 2025). By design, this guarantees that all steps of the Endothelion pipeline can be reproduced in a standardized and automated manner.

Upon cloning the Endothelion repository (https://github.com/TCP-Lab/Endothelion), users can seamlessly retrieve the processed expression tables (i.e., outputs of the x.FASTQ pipeline for all included studies) directly from the corresponding Zenodo repository, just by running the command kerblam data fetch. Then, the full workflow for transportome expression analysis in hCMEC/D3 can be reproduced locally with the single command kerblam run hCMEC_D3. Similarly, the list of GOIs can be independently regenerated by executing the dedicated workflow (kerblam run make_geneset).

## 3 Results

### 3.1 Data collection and filtering of ion channels, transporters, and receptors

To maximize the number of datasets available for our meta-analysis on hCMEC/D3, we performed a systematic search in the NCBI SRA, which yielded eight suitable RNA-Seq studies (Table 1). FASTQ files corresponding exclusively to the untreated control samples (30 paired-end runs in total) were retrieved and reanalyzed using our standardized pipeline, x.FASTQ, as described in the Materials and Methods. Normalized expression matrices, expressed as $\log_{2}\left(\text{TP}M+1\right)$ , were subsequently filtered to retain 672 genes of interest (GOIs) comprising ion channels, transporters, and receptors. Herein, the whole channelome was included, while we excluded secondary transporters involved in the transport of molecules other than inorganic ions, i.e., all those exploiting the ion gradient to transport organic solutes (i.e., amino acids and derivatives, sugars, carbohydrates, nucleosides and nitrogenous bases, vitamins), enzyme cofactors, drugs and xenobiotics, neurotransmitters, inorganic gases. Instead, with regards to receptors (GPCRs and RTKs), which play a key role in the acute and dynamic regulation of cerebral vascular function as they rapidly respond to neurotransmitters and neuromodulators (e.g., ATP, adenosine, and histamine) as well as to growth factors (e.g., vascular endothelial growth factor or VEGF), we focused on those widely described in the literature for their key role during NVC and therefore control of vascular tone but also of BBB permeability in response to physiological or pathological stimuli.

### 3.2 Gene expression profiling across datasets

For each GOI, the mean expression level across the n=8 independent RNA-Seq datasets was calculated along with its associated uncertainty—expressed as a 95% confidence interval—reflecting the variability among datasets. A threshold of TPM ≥1 was applied to GOI’s expression profile to define genes as expressed. Figures 1–3 show only the expressed genes, divided according to their functional group (transporters, ion channels, and receptors). Despite the genetic variability that can affect cell lines, we observed a high level of concordance among the various datasets, suggesting a conserved transcriptional signature and confirming the comparability of the expression datasets after our standardization procedure (Figure 1).

### 3.3 Global expression pattern and functional implications

#### 3.3.1 Ion transporters

Among the most highly expressed ion transporter genes, several subunits of the mitochondrial F-type ATPase complex (e.g., ATP5F1A, ATP5F1B, ATP5MC1, *etc.*) were consistently detected across all datasets (Figure 2; Supplementary Table S2). This reflects the high metabolic demand of cECs, which rely on oxidative phosphorylation to fulfill their high ATP demand (Lai et al., 2023; Shao et al., 2025; Zou et al., 2025). Furthermore, our transcriptomic analysis revealed the expression of multiple genes involved in intracellular Ca^2+^ homeostasis, including *ATP2A2* (SERCA2), *ATP2B1* and *ATP2B4* (PMCA1 and PMCA4), and *ATP2C1* (SPCA1). These genes support active Ca^2+^ clearance across the ER, plasma membrane, and Golgi, respectively (Moccia et al., 2002b; Berra-Romani et al., 2019b; Alexander et al., 2023a). By contrast, we also detected low levels of *ATP2A1* (SERCA1), which is the isoform typically associated with skeletal muscle (Periasamy and Kalyanasundaram, 2007). Similarly, genes showing consistently low expression levels across all datasets include the copper-transporters *ATP7A*, which is widely expressed with low tissue-specificity (Samimi et al., 2003; Niciu et al., 2007; Sudhahar et al., 2020; Ash et al., 2021), and *ATP7B*, which is involved in copper homeostasis in liver (Pierson et al., 2018), and the Na^+^/H^+^ exchangers (*NHE*) 3 and 5, typically enriched in kidney and gastrointestinal tract (Gerbino et al., 2025), and brain tissue (Diering et al., 2009), respectively. Furthermore, sodium/calcium exchanger 1 (*SLC8A1*), which has recently been identified at the protein level in and associated with the onset of the intracellular Ca^2+^ oscillations elicited by removal of extracellular Na^+^ (0 [Na^+^]_o_) in hCMEC/D3 cells (Brunetti et al., 2025), was also found at rather low levels across all datasets.

#### 3.3.2 Ion channels

As showed in Figure 3 and Supplementary Table S3, our transcriptomic analysis detected several Cl^−^-permeable channels among the most highly expressed ion channel-related genes highly, including: the Chloride Intracellular Ion Channel family (CLIC1, CLIC4), Chloride Nucleotide-Sensitive Channel 1A (CLNS1A), Voltage-Dependent Anion-Selective Channel isoforms (VDAC1-3), Leucine-Rich Repeat-Containing 8A (LRRC8A), suggesting a key role in intracellular Cl^−^ regulation and cell volume control, which are essential processes for maintaining BBB integrity and regulating the driving force promoting extracellular Ca^2+^ entry (Nilius and Droogmans, 2001; Alexander et al., 2023b; Liu et al., 2024; Tian et al., 2025).

Despite their functional relevance in mouse brain capillary ECs (Longden et al., 2016), our transcriptomic analysis revealed the low to moderate expression of several K^+^ channels. Notably, we found the expression of the intermediate conductance Ca^2+^-activated channel gene (*KCNN4)* (Figure 3; Supplementary Table S3 **)**, previously detected in mouse arteriolar but not capillary ECs (Longden et al., 2017), which contributes to EDH (Earley, 2011). We also found the transcript for the inwardly-rectifying K_IR_2.1 channel (*KCNJ2*), which senses the modest increase in extracellular K^+^ caused by neuronal activity and thereby initiates EDH (Longden et al., 2017), expressed across all datasets, while K_IR_6.1 (*KCNJ8*), which is a critical subunit for ATP-dependent K^+^ channels and is critical to CBF control in mouse brain microcirculation (Sancho et al., 2022), was undetectable (Figure 3; Supplementary Table S3). On the contrary, we found the transcript for K_Na_1.2 channels, which has never been reported in vascular endothelium previously, as well as BK_Ca_ channels, which is usually expressed in vascular smooth muscle cells (Longden et al., 2016) (Figure 3; Supplementary Table S3). Moreover, we found that hCMEC/D3 cells express the transcripts encoding for connexin Cx43, Cx45 and Cx47, which may form the inter-endothelial gap junctions that enable EDH to electrotonically spread towards the upstream arterioles (Krolak et al., 2025) (Figure 3; Supplementary Table S3). Noteworthy, we also found transcripts encoding for the voltage-gated K^+^ channels K_V_4.1 subunit and its auxiliary KChIP2 (*KCNIP2)* and KChIP3 (*KCNIP3*) subunits (Alexander et al., 2023b), and for the polymodal two-pore domain K^+^ channels, TREK-1 (*KCNK2*) and TWIK-2 (*KCNK6*) (Avalos Prado et al., 2022) (Figure 3; Supplementary Table S3). Interestingly, the regulatory subunits of K_V_1 and K_V_2 channels (*KCNAB2* and *KCNAB3*) were detected, even though the transcripts of the corresponding pore-forming channels were not identified (Figure 3; Supplementary Table S4). Similarly, auxiliary subunits of voltage-gated Ca^2+^ channels (i.e., *CACNB1*, *CACNB3*, *CACNA2D1*, *CACNA2D4*, *CACNG6*, and *CACNG8*) were found, but not their pore-forming α subunits (Figure 3; Supplementary Table S4).

Our transcriptomic analysis showed also the low to moderate expression of different Na^+^-permeable channels in hCMEC/D3 cells, including the Na^+^ leakage channel (*NALCN*), which may contribute to maintain the resting V_M_ (Soda et al., 2024a), the hyperpolarization-activated cyclic nucleotide-gated channels 2/3 (*HCN2; HCN3*) (Figure 3; Supplementary Table S3), which may help repolarizing the V_M_ following EDH (Garcia and Longden, 2020), and the α-subunits of several voltage-gated Na^+^ channels (i.e., Na_V_1.5, Na_V_1.6 and Na_V_1.7 along with their ancillary β1 subunit) (Figure 3; Supplementary Tables S3, S4). Moreover, we found transcript of several non-selective cation (NSC) channels, such as the epithelial Na^+^ channel subunits, α and δ (α-ENaC/*SCNN1A* and δ-ENaC/*SCNN1D*), which were recently found to promote CBF recover to the baseline in the mouse brain microcirculation (Duncan et al., 2020; Lim et al., 2024), and TRPV4, already identified at protein level in hCMEC/D3 cells, where it shapes the Ca^2+^ response to arachidonic acid (Berra-Romani et al., 2019a) (Figure 3; Supplementary Table S3). In addition to TRPV4, we confirmed the expression of TRPV1 and TRPV2 transcripts (Luo et al., 2020) (Figure 3; Supplementary Table S3). Other members of the TRP family were identified across the datasets, including TRPC1 and TRPC4, TRPM2, TRPM4 and TRPM7 (Figure 3; Supplementary Table S3).

The mechanosensitive cation channel Piezo1 stands out among the most highly expressed genes across the datasets (Figure 3; Supplementary Table S3), in line with recent studies demonstrating its contribution to fine-tune the hemodynamic response in the mouse brain microcirculation (Lim et al., 2024). In addition, herein we revealed the presence of other putative mechanosensors at the plasma membrane, including TACAN (*TMEM120A*) (Niu et al., 2021; Kang and Lee, 2024), TMC7 (Zhang et al., 2024), TMEM63A and TMEM63B (Zheng et al., 2023), although the precise functions of these channels in ECs remain largely unexplored. In addition, we also identified transcripts for PKD1 and PKD2, which respectively encode polycystin-1 (TRPP1) and polycystin-2 (TRPP2), which may also play a role in vascular mechano-transduction (Solano et al., 2025).

As shown in Figure 3 and Supplementary Table S3, we also reported the expression of several intracellular Ca^2+^-releasing channels in hCMEC/D3 cells. Notably, we detected transcripts encoding for inositol 1,4,5-trisphosphate (InsP_3_) receptors (InsP_3_Rs), which mediate ER Ca^2+^ release, namely, *ITP*_*3*_ *R1*/InsP_3_R1, *ITP*_*3*_ *R2*/InsP_3_R2, and *ITP*_*3*_ *R3*/InsP_3_R3. We also confirmed the expression of two-pore channels (*TPCN1*/TPC1 and *TPCN2*/TPC2) and TRPML1 (*MCLON1*), which were already found to promote lysosomal Ca^2+^ in hCMEC/D3 cells (Berra-Romani et al., 2020; Brunetti et al., 2024a). By contrast, transcripts for ryanodine receptors (RyRs) were not detected, as previously shown in hCMEC/D3 cells (Zuccolo et al., 2019). In addition, we identified expression of *TMEM38A* and *TMEM38B* (Figure 3; Supplementary Table S3), which encode the trimeric intracellular cation channels TRIC-A and TRIC-B, known to support ER Ca^2+^ homeostasis (Zhou et al., 2014). We also found transcripts for a number of putative ER Ca^2+^ leak channels, including TMBIM1, TMBIM4, TMBIM6, TMEM109 (Mitsugumin 23), PSEN1 (Presenilin 1), and TMCO1 (Tu et al., 2006; Liu, 2017; Bouron, 2020) (Figure 3; Supplementary Table S3).

Moreover, the high expression of mitochondria-associated proteins (e.g., the Growth Hormone Inducible Transmembrane protein, GHITM, and the Bax inhibitor 1, TMBIM6) reflects the elevated metabolic demands and stress response capabilities of brain cECs (Figure 3; Supplementary Table S3) (Kim et al., 2008; Oka et al., 2008; Austin et al., 2022; Patron et al., 2022). Furthermore, genes involved in ER protein trafficking (e.g., SEC61A1) are consistent with the scenario of an active intracellular signaling environment (Nishi et al., 1998; Lang et al., 2011; Venturi et al., 2011), potentially linked to tight regulation of protein synthesis.

Overall, these findings suggest that hCMEC/D3 cells express a specialized, although selective, repertoire of ion channels. Notably, several of the detected transcripts were expressed at relatively low levels, which is consistent with their primary roles in other tissues or specialized cell types. For instance, our transcriptomic analysis revealed the expression of transcripts coding for the α subunits of voltage-gated Na^+^ channels, such as *SCN9A* and *SCN8A*, which are predominantly expressed in neurons, or *SCN5A* in cardiac myocytes, and for the Ca^2+^-permeable channels CATSPER1/2, which are essential components of sperm-specific Ca^2+^ signaling (Alexander et al., 2023a).

#### 3.3.3 Membrane receptors

We detected the transcripts of both ionotropic (i.e., P2X4, P2X5 and P2X7) (Scarpellino et al., 2022) and metabotropic (i.e., P2Y2 and P2Y11) (Moccia et al., 2001; Berra-Romani et al., 2008) purinergic receptors (Figures 3, 4; Supplementary Tables S3, S5), consistently with a previous report (Bintig et al., 2012). Similarly, we confirmed the expression of adenosine receptor-related genes (*ADORA1, ADORA2A, ADORA2B),* which were also previously detected and shown to modulate permeability in hCMEC/D3 cell monolayers (Bader et al., 2017). Similarly, we confirmed the expression of the transcript for histamine receptor H1 (*HRH1*) (Figure 4; Supplementary Table S5), a GPCR that elicits intracellular Ca^2+^ oscillations and NO release induced by both histamine and 0 [Na^+^]_o_ in hCMEC/D3 cells (Berra-Romani et al., 2020; Brunetti et al., 2025). Interestingly, we detected the GABA_A_R ε subunit (*GABRE*) (Figure 3; Supplementary Table S3) and GABA_B_R subunit 1 (*GABBR1)* (Figure 4; Supplementary Table S5), which contribute to support GABA-induced Ca^2+^ signals in hCMEC/D3 cells (Negri et al., 2022). Conversely. we did not detected the expression of multiple additional subunits for the ionotropic GABA_A_ and metabotropic GABA_B_ receptors that were previously detected at protein level in hCMEC/D3 cells (Negri et al., 2022).We also found transcripts for the nicotinic acetylcholine receptors (nAchRs) subunit α5 and β1 (*CHRNA5* and *CHNRB*, respectively) and for *CHRFAM7A*, a negative regulator of the nicotinic acetylcholine receptor α7 (nAChR7), whereas the transcript for *CHRNA7* was not found (Figure 4; Supplementary Table S3). On the contrary, we did not detect the transcript encoding for the M5 muscarinic acetylcholine receptor (*CHRM5*), which has previously been identified in hCMEC/D3 cells (Zuccolo et al., 2019). Moreover, our transcriptomic analysis showed the expression of α1-, β1- and β2- adrenergic receptors (*ADRA1B*; *ADRB1*; *ADRB2*) (Figure 4; Supplementary Table S5). Interestingly, we did not find transcripts for both ionotropic and metabotropic glutamate receptors, respectively the N-methyl-D-aspartate (NMDA) receptors GluN1 (*GRIN1*), GluN2C (*GRIN2C*) and GluN3B (*GRIN3B*), and the Group 1 metabotropic receptors (*GRM1* and *GRM5*), which were previously detected in hCMEC/D3 cells (Negri et al., 2020; Negri et al., 2021a).

Growth factor-related RTKs are key regulators of vascular biology and play critical roles in the development, maintenance, and dynamic modulation of the BBB (Obermeier et al., 2013). Our transcriptomic analysis revealed the expression of the transcripts for FGFR-1 (fibroblast growth factor receptor 1) and FGFR-3, IGF1R (insulin-like growth factor 1 receptor), EGFR (epidermal growth factor receptor), PDGFRB (platelet-derived growth factor beta), VEGFR-1 (vascular endothelial growth factor receptor-1 or Flt-1) and VEGFR-2 (or KDR) family members (Figure 4; Supplementary Table S6). Collectively, these findings provide new insights into the repertoire of ionotropic receptors, GPCRs, and RTKs expressed in hCMEC/D3 cells, which may serve as a reference to investigate the complex signaling network that regulates the structural and functional properties of the BBB, making them potential therapeutic targets in a range of neurological diseases.

## 4 Discussion

In this study we investigated the expression pattern of 672 genes by exploiting datasets from eight different RNA-Seq analysis conducted on hCMEC/D3 cells. Most of the knowledge about the ion transport mechanisms and the GPCR signaling toolkit of the BBB has been generated by *in vivo*, *ex vivo*, or *in vitro* studies carried out on mouse microcirculation. Therefore, profiling the expression of the ion transport mechanisms, GPCRs, and RTKs of the human BBB may aid in delineating how the human brain cECs maintain the ionic milieu that sustains neuronal activity and how they sense and transduce neuronal activity and subtle alterations in the physicochemical properties of local microenvironment, such as extracellular acidosis or functional hyperemia. The hCMEC/D3 cell line represents the most widespread model of human BBB (Weksler et al., 2013; Helms et al., 2016; Qi et al., 2023). So far, the great majority of studies regarding the endothelial transport across the human BBB focused on the two major classes of drug and nutrient transporters: adenosine triphosphate binding cassette (ABC) and solute carrier (SLC) transporters (Okura et al., 2014; Higuchi et al., 2021; Kumabe et al., 2025). Therefore, we have only considered the genes encoding for ion channels, ion transporters, membrane ATPases, GPCRs, and RTKs, as they are altogether responsible for the finely tuned regulation of the major processes regulated by the BBB: ion transport, changes in BBB permeability, and CBF regulation. Furthermore, if available, we correlated functional expression data previously reported on hCMEC/D3 cells, with the transcriptional expression profile emerging from the RNA-Seq datasets. For space constraints, we focus on transporters, ion channels, GPCRs, and RTKs for which a functional role within the NVU is well understood (Garcia and Longden, 2020).

We anticipate that, although the hCMEC/D3 cell line represents the most widespread model of human BBB (Weksler et al., 2013; Helms et al., 2016; Qi et al., 2023), it presents intrinsic limitations that should be considered when interpreting transcriptomic and functional data. First, we considered transcriptomic data obtained from hCMEC/D3 cells cultured under static conditions, therefore lacking the shear stress generated by blood flow, which is a key physiological stimulus known to modulate endothelial gene expression, barrier properties, and mechanosensitive signaling pathways (Cucullo et al., 2011; DeStefano et al., 2017; Choublier et al., 2022; DeOre et al., 2022). Second, as an immortalized cell line, hCMEC/D3 cells may exhibit alterations in the differentiation state, metabolic features, and membrane transporter expression as compared to primary human brain microvascular endothelial cells (Weksler et al., 2013; Qi et al., 2023; Adams et al., 1989). Finally, this model does not recapitulate the multicellular complexity of the NVU, as it lacks supporting cell types such as astrocytes, pericytes, and neurons, which are essential for inducing and maintaining full BBB phenotype and function (Weksler et al., 2013; Qi et al., 2023).

### 4.1 The ion transport machinery responsible for the brain interstitial fluid (ISF) secretion

Ion and water transport across the BBB contributes to maintain the appropriate volume and ionic composition of the ISF. The ISF continuously interchanges with the cerebrospinal fluid due to a convective bulk flow movement facilitated by water channels, known as aquaporin-4 (AQP4) (Iliff et al., 2012; Mestre et al., 2018), which enable water to follow the osmotic gradients mainly determined by the flow of Na^+^ and Cl^−^. Surprisingly, no aquaporins have been identified to date in human brain ECs (Adiele et al., 2025); however, here we highlighted for the first time the expression of AQP3 and AQP5 in the hCMEC/D3 cell line (Supplementary Table S3). AQP3 and AQP5 are also able to conduct hydrogen peroxide (H_2_O_2_) and, by doing so, they regulate redox signaling (Tamma et al., 2018) and trigger H_2_O_2_-dependent endothelial Ca^2+^ signals (Martinotti et al., 2019). Given that astrocytic endfeet AQP4 is the main responsible for water transport in the human brain, the expression of AQP3 and AQP5 in hCMEC/D3 cells may reflect complementary, non-canonical functions of aquaporins, potentially dependent on the *in-vitro* cellular state. Indeed, both AQP3 and AQP5 have been previously detected in cultured vascular and lymphatic ECs (da Silva et al., 2018; Elkhider et al., 2020; Yang et al., 2024), astrocytes and neurons (Yamamoto et al., 2001). Their expression may be associated with the modulation of redox-sensitive pathways (Miller et al., 2010; Hara-Chikuma et al., 2015), support cell-volume regulation (Kida et al., 2005; Chen et al., 2011), migratory dynamics (Rose et al., 2021; Silva et al., 2022).

The mechanism of Na^+^ and Cl^−^ secretion across the BBB has long been matter of intense investigation in rodent microcirculation (Mokgokong et al., 2014; O'Donnell, 2014; Kadry et al., 2020): Na^+^ and Cl^−^ enter the BBB primarily via the Na^+^-K^+^-Cl^−^ cotransporter (NKCC), which is located on the abluminal membrane of cECs, while Na^+^ primarily leaves via the Na^+^,K^+^-ATPase, which is located on the luminal side of brain capillaries (O'Donnell, 2014). The exit route for Cl^−^ is unknown, but it is likely mediated by a Cl-permeable channel and a K^+^/Cl^−^ cotransporter (KCC) (O'Donnell, 2014). The unbalance in these transport activities could lead to pathological outcome, as in the case of ischemic stroke, in which increased activity of BBB luminal Na^+^ transporters results in ‘hypersecretion’ of Na^+^, Cl^−^, and water into the brain interstitium (O'Donnell, 2014). Additional Na^+^-dependent secondary active transport systems, which are located in the abluminal membrane of mouse brain cECs, include: Na^+^/H^+^ exchanger (NHE), Na^+^/HCO3^-^ cotransporter (NBC), Na^+^/Ca^2+^ exchanger (NCX), and Cl^−^/HCO3^-^ exchanger (Mokgokong et al., 2014; O'Donnell, 2014; Kadry et al., 2020). Furthermore, the abluminal side of the BBB expresses several K^+^-permeable channels, e.g., the inwardly rectifying K_IR_2.1 and the ATP-dependent K_IR_6.1 channels, which mediate K^+^ removal from brain parenchyma (O'Donnell, 2014; Kadry et al., 2020). The plasma membrane Ca^2+^-ATPase (PMCA) has been detected on the luminal side and may contribute to removing excess Ca^2+^ from the brain ISF into the blood (Manoonkitiwongsa et al., 2000). This polarized arrangement of pumps, exchangers, and ion channels is crucial for the BBB to maintain the ionic milieu required for neuronal function and synaptic activity, to prevent shifts in the ISF pH, and regulate intracellular volume (O'Donnell, 2014; Kadry et al., 2020).

In the present investigation, we screened the hCMEC/D3 RNA-Seq datasets for the transcriptional expression of transporters, ATPases and ion channels potentially involved in the production and regulation of the ISF, as reported in Supplementary Tables S2, S3. The transcripts encoding all the components of the ion transport machinery described above are expressed in hCMEC/D3 cells, thereby suggesting that the mechanisms responsible for ISF secretion are similar to those described in mouse brain microcirculation. Intriguingly, the NKCC1 protein has been detected in hCMEC/D3 cells (Luo et al., 2018) and suggested to play a role in V_M_ modulation (Soda et al., 2024a). Furthermore, RT-qPCR confirmed that NHE1, NHE5, and NBCn1 transcripts are expressed in hCMEC/D3 cells (Luo et al., 2018). We also detected mRNA for the voltage-gated H^+^ channel (H_V_1), which has not been reported previously before in vascular ECs. H_V_1 channels may be critical for intracellular pH regulation by mediating H^+^ efflux during biochemical reactions that acutely acidify the cytosol (Luo et al., 2021). This transcriptomic profile provides a framework for understanding the movement of ions into and out of the human brain endothelium at the BBB. Studies on mouse brain microcirculation revealed that brain disorders, such as traumatic brain injury, epilepsy, ischemia, hypoxia, and neurodegenerative disorders, may cause significant changes in the expression of the ion transport machinery at the BBB (Abbott et al., 2010; Kadry et al., 2020). Therefore, this investigation may be useful to effectively target the human NVU and thereby attenuate the alterations in the ionic composition of the ISF associated with these disorders.

### 4.2 Electrogenesis: resting V_M_, EDH and other ion channels potentially involved in the regulation of V_M_ during agonist stimulation

The repertoire of ion channels that regulate the V_M_ of hCMEC/D3 cells, both under resting conditions and in response to extracellular stimuli, remains largely uncharacterized (Bader et al., 2017; Berra-Romani et al., 2023). It was recently shown that hCMEC/D3 cells exhibit a depolarized resting V_M_ (∼-16 mV), largely driven by a Na^+^ leakage and basal Cl^−^ permeability (Soda et al., 2024a). Basal Na^+^ permeability has been repeatedly reported in microvascular ECs, which may therefore display a depolarized V_M_ (Voets et al., 1996; Nilius and Droogmans, 2001; Moccia et al., 2002a). Our transcriptional analysis showed that hCMEC/D3 cells express the NALCN channel, which is a widely expressed Na^+^-selective channel in the CNS. NALCN channels mediates the Na^+^ leak in neurons, which results in a resting V_M_ that is a more depolarized value than the K^+^ equilibrium potential (E_K_) (Monteil et al., 2024). This is the first evidence that the NALCN channel is expressed in hCMEC/D3 cells, where it may contribute to the background Na^+^ conductance that maintains the resting V_M_ well above E_K_ (Soda et al., 2024a). Additionally, NALCN channels could underlie the basal Na^+^ permeability that has been described in ECs from multiple species and vascular districts (Manabe et al., 1995; Voets et al., 1996; Park et al., 2000; Moccia et al., 2002a). In accordance with this hypothesis, the background Na^+^ current in vascular ECs is inhibited by extracellular Ca^2+^ (Manabe et al., 1995; Park et al., 2000; Moccia et al., 2002a), which is a feature of NALCN channels (Monteil et al., 2024). The basal Cl^−^ conductance that contributes to the resting V_M_ in ECs may be mediated by either volume-sensitive (Voets et al., 1996) or Ca^2+^-activated Cl^−^ channels (CaCCs) (Moccia et al., 2002a). All the members of the LRCC8/Swell1 family, which mediate volume-regulated anion channels (VRACs) (Karakas et al., 2025), are expressed in hCMEC/D3 cells (Supplementary Table S3). However, preliminary analysis suggests that CaCCs, rather than VRACs, mediate the basal Cl^−^ permeability in hCMEC/D3 cells (Soda et al., 2024a). TMEM16A is the most abundant CaCC isoform expressed in mouse brain microvascular ECs (Suzuki et al., 2020). However, TMEM16A is absent in hCMEC/D3 cells, which display the transcripts for TMEM16F, TMEM16G, and TMEM16H (Supplementary Table S3). In addition, they express Bestrophin 1, which primarily functions as CaCC in retinal pigment epithelium, but is not typically associated with ECs (Owji et al., 2021).

The *KCNJ2* gene, which encodes for the inwardly-rectifying K_IR_2.1 channel (Longden et al., 2017), is also expressed in the RNA-Seq datasets analyzed in the present study. The expression of K_IR_2.1 channels in human cerebrovascular ECs may have therapeutic relevance as studies conducted in the mouse microcirculation suggest that they are critical mediators of NVC and represent promising targets for the treatment of neurological disorders (Negri et al., 2021d). K_IR_2.1 channels may represent a mechanism by which human brain cECs sense neuronal firing and signal to upstream parenchymal arterioles the increased demand for blood supply (Longden et al., 2017). Therefore, future work should assess whether they are activated by the modest increase in extracellular K^+^ concentration that occurs during neuronal activity, as shown in mouse capillary ECs. In this view, hCMEC/D3 cells express the *KCNN4* gene, encoding the IK_Ca_/K_Ca_3.1 channels that have not been detected at the mouse BBB (Longden et al., 2017). IK_Ca_/K_Ca_3.1 channels open in response to an increase in [Ca^2+^]_i_ during agonist stimulation (e.g., neurotransmitters, see below), thereby promoting endothelial-dependent hyperpolarization (EDH) (Earley, 2011). K^+^ efflux through IK_Ca_/K_Ca_3.1 channels, along with K^+^ released during neuronal firing, may then activate the endothelial K_IR_2.1 channel to boost EDH, as reported in mouse brain arterioles that highly express the *KCNN4* gene (Longden and Nelson, 2015; Longden et al., 2017). EDH, in turn, spreads to upstream arterioles through inter-endothelial gap junctions, thereby promoting vasorelaxation and increasing blood supply to downstream capillaries (Longden et al., 2017). In the rodent brain microcirculation, gap junctions are primarily made by connexin (Cx) 37 (Cx37), Cx37, Cx40, and Cx45 (Avila et al., 2011; Krolak et al., 2025). Here, we found that hCMEC/D3 cells express the transcripts encoding Cx43, Cx45 and Cx47, which is a Cx isoform typically associated with lymphatic endothelium (Ferrell et al., 2010; Davis et al., 2024). Intriguingly, hCMEC/D3 cells also express HCN2/3 channels (Supplementary Table S3), which are activated by membrane hyperpolarization at voltages to around −50 mV and may help repolarizing the V_M_ following EDH (Garcia and Longden, 2020). In agreement with transcriptomic (Garcia and Longden, 2020) and functional (Longden et al., 2017) data reported from the mouse brain BBB, hCMEC/D3 cells do not express the *KCNN3* gene (Supplementary Table S3), encoding the SK_Ca_/K_Ca_2.3 channels.

As outlined in the next section, hCMEC/D3 cells present many NSC channels that could be activated by subtle changes in the intra- or extracellular microenvironments. When NSC channels carry a high fractional Ca^2+^ current and are physically coupled to SK_Ca_ channels, they may cause endothelial hyperpolarization (Mehrke et al., 1991). Alternatively, they lead to endothelial depolarization (Marchenko and Sage, 1993; Wu et al., 2000; Moccia et al., 2004; Lim et al., 2024), potentially triggering an unexpected bioelectrical response in hCMEC/D3 cells. The transcriptomic analysis of all the eight RNA-Seq databases showed that these cells express the α-subunits, which form the ion-conduction pores, of several voltage-gated Na^+^ channels, i.e., Na_V_1.5, Na_V_1.6, and Na_V_1.7 (Supplementary Table S3) along with their ancillary β1 subunit (Supplementary Table S4). Na_V_1.5 and Na_V_1.7 have previously been detected in mouse endothelial cells from the skin vasculature (Rice et al., 2015) and human umbilical vein endothelial cells (Andrikopoulos et al., 2011), in which they support vascular endothelial growth factor-induced angiogenesis. Additionally, hCMEC/D3 cells express the auxiliary subunits of both voltage-gated K^+^ and Ca^2+^ channels (Supplementary Table S4), such as β, α2δ, and γ subunits, although the pore-forming K_V_1 and Ca_V_ isoforms are absent. However, emerging evidence suggests that auxiliary subunits of Ca_V_1.2 channels can engage in signaling pathways independent of their interaction with ion channels (Risher and Eroglu, 2020; Vergnol et al., 2022). For instance, the Cavβ3 subunits, which is expressed in hCMEC/D3 cells (Supplementary Table S4), regulates the BBB permeability in the mouse microcirculation (Martus et al., 2024). Conversely, hCMEC/D3 cells express the transcripts for the molecular components of the A-type K^+^ current, i.e., the pore forming K_V_4.1 subunit and its auxiliary KCHIP3 (*KCNIP3*) subunits (Alexander et al., 2023b). This current is likely to be inactivated at the resting V_M_, but could de-inactivate following agonist-induced hyperpolarization, thereby contributing to modulating the bioelectrical activity of hCMEC/D3 cells.

A number of K^+^ channels that have never been detected in vascular ECs could act as a brake on depolarization. Our transcriptomic analysis showed that hCMEC/D3 cells express Na^+^-activated K^+^ channels (K_Na_1.2 or Slo2.1), which may be activated by Na^+^ entry through NSC channels, and BK_Ca_ channels (Supplementary Table S3), which may be activated by both Ca^2+^ entry and membrane depolarization (Alexander et al., 2023b). Unlike SK_Ca_ channels, BK_Ca_ channels are generally not expressed in vascular endothelial cells; however, they are abundantly present in vascular smooth muscle cells, in which they promote vasoconstriction (Longden et al., 2016). Similarly, K_Na_1.2 channels have never been reported in vascular endothelium. The finding that hCMEC/D3 cells undergo hyperpolarization upon a reduction in extracellular Na^+^ concentration suggests that K_Na_1.2 channels are not activated by the basal Na^+^ entry (Soda et al., 2024a). Yet, they could be gated by the massive Na^+^ entry that occurs upon activation of NSC channels (Kaczmarek, 2013), thereby contributing to restoring the resting V_M_.

### 4.3 The intracellular Ca^2+^ toolkit

An increase in endothelial [Ca^2+^]_i_ is critical to fine tune several functions of the BBB, including the regulation of paracellular permeability through the modulation of junctional and cytoskeletal proteins (De Bock et al., 2013), the hemodynamic response to synaptic activity (Negri et al., 2021d; Moccia et al., 2022), and sprouting angiogenesis (Lambrichts et al., 2025). Here, we investigated the expression of the multifaceted components of the Ca^2+^ signaling toolkit in hCMEC/D3 cells. We focused on both the Ca^2+^ pumps and exchangers that maintain and restore resting Ca^2+^ levels following agonist stimulation (Supplementary Table S2) and on the ion channels that generate intracellular Ca^2+^ signals with specific spatio-temporal profiles (Supplementary Table S3), thereby driving a wide variety of cellular responses (Bintig et al., 2012; Berra-Romani et al., 2020; Berra-Romani et al., 2023; Brunetti et al., 2025; Soda et al., 2025).

Our transcriptional analysis detected specific subunits of plasma membrane Ca^2+^ ATPase (PMCA1 and PMCA4), sarco-endoplasmic reticulum Ca^2+^-ATPase (SERCA2), and Na^+^/Ca^2+^ exchanger (NCX1) (Supplementary Table S2). Interestingly, we have recently demonstrated that the NCX1 protein is expressed in hCMEC/D3 cells (Brunetti et al., 2025), whereas a comprehensive RT-qPCR analysis has previously confirmed the presence of the transcripts encoding PMCA1, PMCA4 and SERCA2 (Zuccolo et al., 2019). The endothelial Ca^2+^ response to chemical stimulation is primarily initiated by Ca^2+^ mobilization from the endoplasmic reticulum (ER), the most abundant endothelial Ca^2+^ reservoir, and maintained by extracellular Ca^2+^ entry across the plasma membrane (Moccia et al., 2023a). Transcript analysis revealed the expression of several candidate ER Ca^2+^ leak channels in hCMEC/D3 cells (Supplementary Table S3): TMBIM2 (Lisak et al., 2015), TMBIM6 (Lisak et al., 2015), and mitsugumin 23 (Venturi et al., 2011). ER Ca^2+^ depletion induced by pharmacological inhibition of SERCA activity could occur through any of these pathways, while TMBIM2 and TMBIM6 may also be expressed in the Golgi apparatus (Lisak et al., 2015). Ca^2+^ release from ER requires agonist binding to GPCRs (mainly coupling to G_q_ protein), or TKRs, activating distinct isoforms of phospholipase C (i.e., respectively, PLCβ and PLCγ). PLC cleaves the membrane phospholipid phosphoinositide 4,5-bisphosphate (PIP_2_) into the second messengers, diacylglycerol (DAG) and InsP_3_, which mobilizes Ca^2+^ by gating InsP_3_Rs on the ER (Moccia et al., 2023a; Moccia et al., 2023b). InsP_3_Rs represent the major family of ER Ca^2+^-releasing channels in the endothelial lineage, as all the known InsP_3_R isoforms, i.e., InsP_3_R1, InsP_3_R2 and InsP_3_R3, are expressed in vascular ECs (Moccia et al., 2023b). Herein, we confirmed that InsP_3_R1, InsP_3_R2 and InsP_3_R3 are also expressed in the hCMEC/D3 cell line (Supplementary Table S3). Interestingly, a previous RT-qPCR analysis failed to detect the transcripts encoding for InsP_3_R1 and InsP_3_R2 (Zuccolo et al., 2019). In contrast, ryanodine receptors (RyRs), are unlikely to participate in Ca^2+^ mobilization from ER in endothelial cells (Thakore and Earley, 2019): none of the three known RyR isoforms (RYR1, RYR2, RYR3) is expressed in the hCMEC/D3 cell line (Supplementary Table S3), as previously confirmed by RT-qPCR (Zuccolo et al., 2019). In addition to ER Ca^2+^ release, another key component shaping endothelial Ca^2+^ dynamics is lysosomal Ca^2+^ release through TPCs, which are gated by the intracellular messenger nicotinic acid adenine dinucleotide phosphate (NAADP) and may be present in two distinct isoforms, i.e., TPC1 and TPC2 (Negri et al., 2021b). Herein, we confirmed that they are both expressed in hCMEC/D3 cells (Supplementary Table S3), as previously demonstrated by RT-qPCR (Zuccolo et al., 2019). The transcriptomic analysis confirms that agonist-induced intracellular Ca^2+^ release in human cerebrovascular endothelial cells is supported by ER Ca^2+^ release through InsP_3_Rs and lysosomal Ca^2+^ mobilization through TPCs, as shown by functional analyses (Berra-Romani et al., 2020; Negri et al., 2022). Interestingly, the dataset analysis also revealed the expression of another lysosomal Ca^2+^ channel, i.e., TRPML1 (Supplementary Table S3), which plays a crucial role in autophagy and endolysosomal trafficking. The endothelial role of TRPML1 is not clear yet (Negri et al., 2021b), but we have recently demonstrated that the TRPML1 protein is expressed in acidic endolysosomal vesicles, contributes to ER Ca^2+^ refilling and supports NO release (Brunetti et al., 2024a).

Agonist-induced extracellular Ca^2+^ entry in ECs mainly occurs through the Store-Operated Ca^2+^ Entry (SOCE) pathway, which is mediated by the interaction between Stromal Interaction Molecule (STIM) and ORAI proteins or members of the Transient Receptor Potential Canonical (TRPC) channels, including TRPC1 and TRPC4 (Moccia et al., 2023a). Our transcriptional analysis revealed that both STIM1 and STIM2, which serve as sensors of the luminal Ca^2+^ concentration, are expressed in hCMEC/D3 (Supplementary Table S3), although previous RT-qPCR analysis failed to detect STIM1 transcripts (Zuccolo et al., 2019). Similarly, we confirmed the previous report that all the ORAI isoforms, i.e., ORAI1, ORAI2 and ORAI3, are expressed in human brain cECs (Supplementary Table S3). It is worth noting that the transcripts encoding for TRPC1 and TRPC4 were present in all the eight datasets analysed (Supplementary Table S3), while it was not detected by RT-PCR analysis (Zuccolo et al., 2019). However, the pharmacological profile of the SOCE machinery in hCMEC/D3 cells suggests the involvement of ORA1 channels rather than TRPC1 (Zuccolo et al., 2019; Negri et al., 2020; Negri et al., 2021a). TRPC1 may be gated by a store-independent mechanism, as it has also been shown to assemble with TRPP2 and function as a mechano-sensor in mouse cerebrovascular endothelium (Berrout et al., 2012). Notably, the transcript for TRPP2 is expressed in hCMEC/D3 cells (Supplementary Table S3).

### 4.4 Ionotropic receptors, GPCRs, and RTKs

It has long been known that the brain microcirculation receives extensive innervation from both local neurons, such as pyramidal neurons and γ-aminobutyric (GABA) interneurons, and subcortical afferents, such as basal forebrain, locus coeruleus, ventral tegmental area, and Raphe (Schaeffer and Iadecola, 2021). Multiple lines of evidence indicate that neuronal activity can stimulate NVC (Negri et al., 2021d; Moccia et al., 2022; Soda et al., 2024b), promote angiogenesis (Li et al., 2018; Agrud et al., 2022), and regulate BBB permeability (De Bock et al., 2013; Kaplan et al., 2020) by directly activating cECs within the NVU. Our transcriptomic analysis revealed that hCMEC/D3 cells express typical endothelial as well as neuronal ionotropic (Supplementary Table S3), GPCRs (Supplementary Table S5), and RTKs (Supplementary Table S6). We detected the presence of the transcripts for the purinergic P2X4, P2X5, and P2X7, which mediate Ca^2+^-permeable non-selective cation channels (Scarpellino et al., 2022), and P2Y2 and P2Y11, which are G_q_PCRs triggering InsP_3_-dependent ER Ca^2+^ release (Moccia et al., 2001; Berra-Romani et al., 2008). This finding is consistent with a previous report demonstrating the expression and functional role of these purinergic receptors, as well as P2Y12, in hCMEC/D3 cells (Bintig et al., 2012). Similarly, we confirmed the presence of the histamine receptor H1 (Supplementary Table S5), which elicits histamine- and 0 [Na^+^]_o_-induced intracellular Ca^2+^ oscillations and NO release (Berra-Romani et al., 2020; Brunetti et al., 2025), as well as of the GABA_A_R ε subunit and GABA_B_ receptor subunit 1, which contribute to support GABA-induced Ca^2+^ signals in hCMEC/D3 cells (Negri et al., 2022). Interestingly, our transcriptomic analysis also showed the expression of the transcripts coding for α1- and α2-adrenergic receptors (Supplementary Table S5), which could be involved in the regulation of the human BBB permeability by catecholamines, as shown in the mouse microcirculation (Urayama et al., 2015). Additionally, hCMEC/D3 cells are equipped with the β1-and β2-adrenergic receptors (Supplementary Table S5), which were shown to modulate the angiogenic activity in the human brain microvascular EC (HBMEC) line (Annabi et al., 2009). Consistent with this evidence, an early study documented the presence of α1-, α2, β1-, and β2-adrenergic receptors in microvascular ECs isolated from three different regions of the human brain (Bacic et al., 1992). Our transcriptomic analysis revealed the presence of the transcript for the glycine receptor (GlyR) β subunit (Supplementary Table S3), which may promote post-ischemic angiogenesis and neurological regeneration in the mouse brain (Xu et al., 2024). We also found transcripts for several nAchRs, such as α5 and β1 (Supplementary Table S3). However, the α5-nAchR subunit only plays a modulatory role and is therefore unlikely to mediate acetylcholine-induced inward currents (Alexander et al., 2023a), whereas it could contribute to cancer growth and angiogenesis by engaging the Smad signaling pathway in a flux-independent manner (Cai et al., 2025). Similarly, the β1 subunit primarily contributes to the muscle nAchR and has never been detected in the brain (Tae and Adams, 2023). Furthermore, hCMEC/D3 cells also express Dupα7, which serves as dominant negative inhibitor of nAchR7 (Dang et al., 2015). These findings are, therefore, consistent with the report that nicotine does not trigger a detectable increase in [Ca^2+^]_i_ in hCMEC/D3 cells (Zuccolo et al., 2019), but additional work is mandatory to assess the functional role of α5-nAchR, β1-nAchR, and Dupα7 at the human BBB. It is likely that these subunits signal in a flux-independent, i.e., metabotropic, manner, as already demonstrated for nAchRs (Montes de Oca Balderas, 2022) and ionotropic glutamate receptors (Brunetti et al., 2024b). Finally, our transcriptomic analysis confirmed the expression of transcripts for adenosine receptors, such as A1, A2a and A2b, which were previously detected and shown to modulate permeability in hCMEC/D3 cell monolayers (Bader et al., 2017). Moreover, the endothelial adenosine receptors could play a role in NVC, by activating K_ATP_ channels and promoting EDH (Mughal et al., 2024a). It should, however, be pointed out that we could not find a significant expression of the transcripts for several GPCRs that were previously detected functionally characterized in hCMEC/D3 cells, including M5 receptors for acetylcholine (Zuccolo et al., 2019), NMDA and Group 1 metabotropic receptors for glutamate (Negri et al., 2020; Negri et al., 2021a), and multiple additional subunits for the ionotropic GABA_A_ and metabotropic GABA_B_ receptors (Negri et al., 2022). The potential reasons for this discrepancy will be discussed below.

Our transcriptomic analysis identified members of the FGFR, IGFR, EGFR, PDGFR, and VEGFR families (Supplementary Table S6), a repertoire that likely reflects the multifaceted signaling environment of the brain endothelium. Several of these receptors have already been linked to BBB biology in experimental models. For example, IGF1R contributes to BBB preservation in mice (Gulej et al., 2024) and enhances endothelial stability through junctional protein upregulation (Higashi et al., 2020). PDGFR beta (PDGR-B) is essential for endothelial–pericyte communication and vascular maturation (Shen et al., 2019), while FGFR1 activation has been associated with improved barrier tightness and permeability control *in vivo* (Chen et al., 2020; Kriauciunaite et al., 2023). VEGF is the master regulator of angiogenesis throughout peripheral circulation (Moccia et al., 2019), including the brain microvasculature (Lange et al., 2016). VEGF regulates microvessel density and endothelial permeability at the BBB, although pathological VEGF signaling may disrupt BBB integrity and cause vascular leakage (Lange et al., 2016). The effect of VEGF on the cerebral microcirculation is primarily mediated by VEGFR-2 (also known as KDR) (Wen et al., 2023), which is abundantly expressed in all the 8 RNA-Seq datasets (Supplementary Table S6). This finding is consistent with recent reports confirming VEGFR-2 expression and signaling in the hCMEC/D3 cell line (Zhang et al., 2019; Sandoval et al., 2025). We also detected transcripts for VEGFR-1 (also known as Flt-1) (Supplementary Table S6), which has been previously described in hCMEC/D3 cells, although its functional role remains unclear (Teles et al., 2025). In the human brain microcirculation, VEGFR-1 is likely to exert protective roles against inflammatory cues or stress-induced disruption of the BBB, as reported in other cellular (Salmeri et al., 2013) and animal (Schreurs et al., 2012) models. Finally, we confirmed the expression of EGFR, which has recently been shown to regulate the permeability of hCMEC/D3 cell monolayers by recruiting the c-Jun N-terminal kinase signaling pathway (Chen et al., 2015). Collectively, the detection of this RTK subset highlights that hCMEC/D3 cells not only present barrier-related features but also possess the molecular machinery to respond to angiogenic and trophic cues, important to maintain BBB integrity.

### 4.5 Sensing changes in extra/intracellular environments

Brain endothelial cells must constantly adapt to shear stress, oxidative stress, and changes in the ionic composition of the ISF. Our transcriptomic analysis revealed that, to do so, they may exploit several mechano-sensitive, pH-sensitive and redox-sensitive ion channels. A local increase in CBF is critical for supplying active neurons with oxygen and nutrients (Negri et al., 2021d). The hemodynamic response to neuronal activity also causes a local increase in shear stress that can be detected by brain cECs through mechano-sensitive ion channels. Our transcriptomic analysis revealed that hCMEC/D3 cells express several mechanosensitive NSC channels detected in other EC types (Lim and Harraz, 2024), including (Supplementary Table S3): Piezo1, TRPV4, PKD1 (TRPP1), PKD2 (TRPP1), ENaC, and TRPM7. Consistent with this finding, we previously showed that TRPV4 protein is expressed and mediates robust NO release in hCMEC/D3 cells (Berra-Romani et al., 2019a). Moreover, endothelial Piezo1 channels and ENaC have recently been detected in the mouse brain microcirculation (Duncan et al., 2020; Lim et al., 2024). The available evidence suggests that these mechanosensitive NSC channels play distinct roles during NVC: TRPV4-mediated NO release may support endothelium-dependent vasodilation (Diaz-Otero et al., 2019), while Piezo1-and ENaC-mediated depolarization promotes CBF recovery to the baseline (Ashley et al., 2018; Duncan et al., 2020; Lim et al., 2024). At the current stage, it is unpredictable whether PKD1/PKD2 and TRPM7 channels mediate vasorelaxation through NO release or vasoconstriction through membrane depolarization. Intriguingly, hCMEC/D3 cells also express the *TMEM120* gene, encoding the TACAN protein, which were claimed either to serve as mechano-sensitive NSC channels or to modulate Piezo and PKD2 channels (Kang and Lee, 2024). Our *in silico* results also showed the expression of the genes encoding TREK1 channels in human cerebrovascular endothelial cells (Supplementary Table S3). These are polymodal K^+^-selective channels that can integrate mechanical (e.g., membrane stretch), thermal (increase in temperature from ∼17 to ∼40 °C), and chemical (e.g., arachidonic acid, pH, GPCRs) (Avalos Prado et al., 2022). TREK1 channels may support EDH in response to either the initial phase of the hemodynamic response (when the arteriolar-capillary transitional zone undergoes a local increase in shear stress) or downstream of the G_q_PCRs that contribute to NVC.

In addition to being mechano-sensitive, ENaC and TRPM7 channels may play a crucial role in regulating BBB permeability, brain angiogenesis and inflammation, by detecting changes in Na^+^ and Mg^2+^ concentrations in the ISF (Zhu et al., 2018; Maier et al., 2022; Zhang et al., 2022). Particularly, hyponatremia has been reported in epilepsy and cortical spreading depression (Gankam Kengne, 2023), whereas Mg^2+^ deficiency has been reported in Alzheimer’s disease (Barbagallo et al., 2011) and age-related dementia (Ozturk and Cillier, 2006). Future studies are necessary to determine whether the polymodal ENaC and TRPM7 channels serve as mechano- and/or chemo-sensors in human cerebrovascular endothelial cells. The transcriptomic profile also suggests that hCMEC/D3 cells possess a repertoire of pH- and redox-sensitive ion channels, which may enable them to detect chemical changes in the extracellular milieu of the brain. Extracellular acidification may be associated with a variety of neurological disorders, including migraine, traumatic brain injury, ischemic stroke, multiple sclerosis, and epileptic seizures (Cheng et al., 2025). Studies on mouse microcirculation showed that brain microvascular ECs may detect reductions in extracellular pH through ASIC type 1 (ASIC1) (Lin et al., 2014) and TREK-1 channels (Fang et al., 2019), which exert a neuroprotective role by promoting vasodilation. In addition to TREK-1, ASIC1 channels were detected in all the five RNA-Seq datasets analyzed, suggesting that these endothelial pH-sensitive ion channels may also play a role in human brain microvasculature. Intriguingly, both ASIC1 and TREK-1 channels could contribute to promoting endothelial dysfunction upon strong and/or prolonged brain ischemia (Zhang et al., 2020; Zheng et al., 2022). Gene expression analysis showed that hCMEC/D3 cells express several redox-sensitive NSC channels (Negri et al., 2021c; Schaller et al., 2025), such as TRPM2, TRPV1, TRPV2 and TRPV4 (Supplementary Table S3). In addition to TRPV4 (Berra-Romani et al., 2019a), a recent study confirmed that hCMEC/D3 cells express a functional TRPV2 protein, while TRPV1-mediated Ca^2+^ entry was barely detectable (Luo et al., 2020). Consistently, our transcriptomic analysis shows that TRPV2 gene expression is significantly higher than that of TRPV1, although single-cell Ca^2+^ imaging will be necessary to determine whether TRPV1 activation leads to an increase in [Ca^2+^]_i_. Intriguingly, TRPM2 has been shown to be the primary mediator of ROS-dependent BBB degradation in several brain disorders (Ding et al., 2021), including ischemic stroke (Zong et al., 2024) and Alzheimer’s disease (Park et al., 2014). A preliminary investigation confirmed that the TRPM2 protein is expressed and mediates oxidative stress-induced injury in hCMEC/D3 cell monolayers (Huang et al., 2021).

### 4.6 The apparent discrepancy between transcriptomic and functional data

Interestingly, the expression pattern of transporter-related genes (Figure 1) markedly differs from that of ion channel genes (Figure 2). While the majority of transporters exhibit relatively high and consistent expression across datasets, ion channels tend to display lower overall expression and greater inter-sample variability. This pattern suggests a more functionally essential expression of transporters in hCMEC/D3 cells, whereas ion channels may play more context-dependent or specialized roles (see also below). Indeed, transporters are often responsible for constitutive cellular functions, such as nutrient uptake, metabolite exchange, and waste removal. These functions are critical for endothelial homeostasis and are generally preserved across endothelial subtypes and vascular districts. Additionally, the blend of endothelial transporters is critical for controlling the neuronal excitability as well as synaptic signaling. As a result, their transcript levels are typically stable and reproducible, even across datasets generated under differing experimental conditions.

A critical factor contributing to the low apparent transcript levels of many ion channels is that these classes of membrane proteins are often expressed at intrinsically low copy numbers (Grygorczyk et al., 1984; Beck et al., 2011; Lockwich et al., 2011; O'Leary et al., 2013; Thillaiappan et al., 2019; Gorur-Shandilya et al., 2020; Ali Shah and Ou, 2021; von Lindern et al., 2022), yet can exert substantial physiological effects due to high ligand sensitivity, steep dose–response relationships, and strong downstream amplification within signaling cascades. In accord, many ion channels operate in highly localized membrane microdomains, such as caveolae and myo-endothelial gap junctions, where even sparse protein expression is sufficient for robust functional output (Berra-Romani et al., 2013; Pires et al., 2015; Sullivan et al., 2015; Murphy and Sandow, 2019; Ottolini et al., 2020; Jackson, 2022; Moccia et al., 2023b; Huo et al., 2025). Consequently, low transcript abundance does not necessarily reflect limited functional capacity, and the presence of functional receptors or channels can be underestimated when mRNA-based quantification is used as the sole readout. Additionally, several transcripts that have been characterized molecularly and functionally in hCMEC/D3 cells, were undetectable in any of the eight public RNA-Seq datasets examined. These transcripts include several components of the Ca^2+^ handling machinery (Berridge et al., 2003), such as ion channels, e.g., TRPA1 (Berra-Romani et al., 2023; Soda et al., 2025), GABA_A_ (Negri et al., 2022) and NMDA receptors (Negri et al., 2021a), and G_q_PCRs, e.g., P2Y12 receptors (Bintig et al., 2012), M5 muscarinic receptors (Zuccolo et al., 2019), GABA_B_ receptors, and Group 1 metabotropic glutamate receptors (Negri et al., 2020). The mismatch between mRNA abundance and the expression levels of membrane proteins, has long been matter of intense discussion (Misquitta et al., 2006; Vogel and Marcotte, 2012; Li et al., 2020). In the case of components of the Ca^2+^ signaling machinery, this discrepancy likely reflects tight post-transcriptional regulation of mRNA stability (Misquitta et al., 2006; Dragoni et al., 2014; Sobradillo et al., 2014; Zuccolo et al., 2018). Transcripts with low abundance or very short half-lives are rapidly degraded after translation, leading to transient mRNA levels that are difficult to capture, especially in bulk RNA-Seq experiments, where signal averaging further reduces sensitivity to short-lived transcripts (Urich et al., 2012). The discordance between protein levels and transcript levels has already been reported for NMDA receptors (Gazzaley et al., 1996; Awobuluyi et al., 2003), P2Y12 receptors (Rauch et al., 2010; Pavlovic et al., 2020), mGluR5 (Iyo et al., 2010; Fatemi et al., 2013), and GABA_A_ receptors (Murgas et al., 2022). Consistent with this, a recent investigation mining a publicly available RNA-Seq dataset failed to detect the transcripts for NMDARs (Garcia and Longden, 2020), which are known to be expressed and regulate the BBB permeability in the mouse microcirculation (Macrez et al., 2016; Mehra et al., 2020). Beyond biological regulation, technical and methodological factors can further contribute to apparent transcript-protein mismatches. Variations in sequencing depth, strandedness, and reference annotation all affect detection sensitivity for low-abundance or alternatively spliced transcripts. Moreover, library-preparation biases, such as the widespread use of poly(A) selection during mRNA enrichment, can distort transcript representation by preferentially capturing polyadenylated transcripts while underrepresenting non-polyadenylated or atypically-processed mRNAs (Yang et al., 2011; Wehrspaun et al., 2014; Witmer et al., 2024). In addition, when highly expressed genes dominate the sequencing read pool, low-abundance/rare mRNAs may fall beyond detection thresholds. Furthermore, if the transcripts exhibit high sequence similarity to paralogs or pseudogenes, their reads may be difficult to map uniquely to the reference genome. This can lead to them being filtered out during standard bioinformatics analysis, as reported for ribosomal proteins (Tonner et al., 2012). On the contrary, transporter genes, often expressed at moderate-to-high levels, tend to yield more robust and reproducible signals in both bulk and single-cell RNA sequencing datasets. These results highlight the importance of targeted experimental validation, such as qRT-PCR, targeted RNA capture, immunoblotting, immunofluorescence, or functional assays, to confirm transcript presence when public RNA-Seq datasets yield negative results.

## 5 Conclusion

Taken together, these findings suggest that ion transporter, GPCR, and RTK toolkit is a reliable and consistent readout for characterizing the endothelial transcriptome across datasets, albeit ion channel profiles may require careful interpretation and validation, particularly in the context of endothelial functional diversity.

## Data Availability

The original contributions presented in the study are included in the article/Supplementary Material, further inquiries can be directed to the corresponding author.
