# Supplementary material for: The ion transport, GPCR, and RTK toolkit expression in the human cerebrovascular endothelial cell line, hCMEC/D3: an Omics perspective
Source: Front Physiol. 2025 Dec 18;16:1733266. doi: 10.3389/fphys.2025.1733266 (PMC12756094; doi:10.3389/fphys.2025.1733266)
Supplement: Supplementary file 1 [file DataSheet1.docx]

Supplementary Material


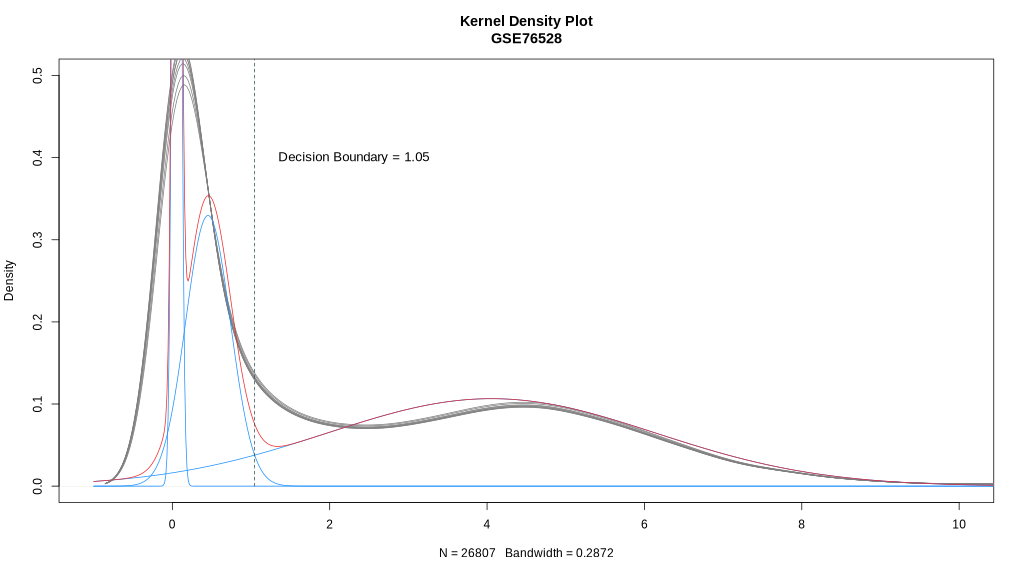


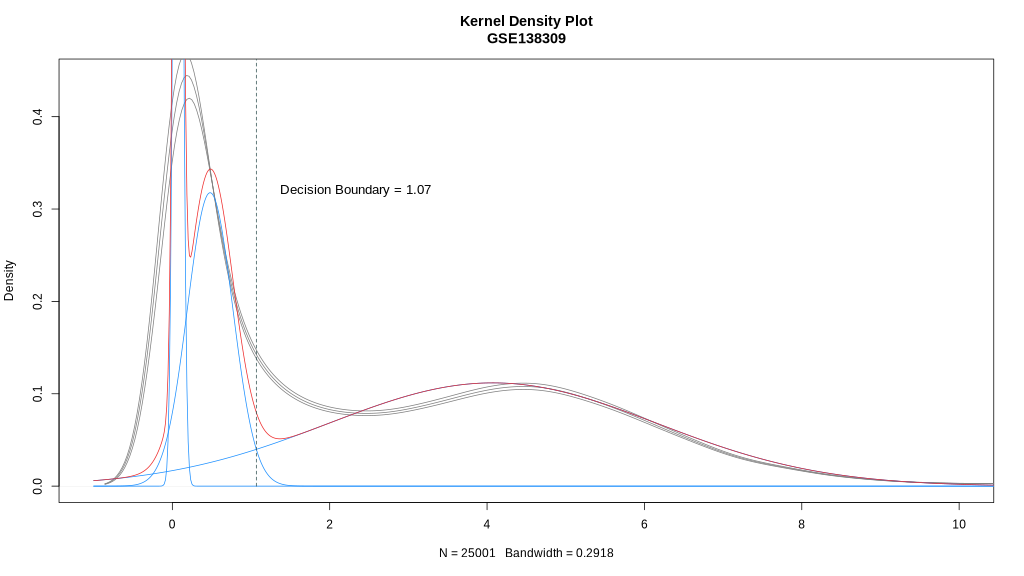


Supplementary Figure 1. Gaussian mixture modelling of gene expression distributions. Kernel density estimate (grey) of the empirical log_2_(TPM + 1) expression values for one representative dataset (zero-count genes removed for improved y-axis scaling), overlaid with the fitted three-component Gaussian mixture model. The three individual Gaussian components are shown in blue: (i) a near-zero component capturing null or quasi-null expression values, (ii) a low-abundance/background component, and (iii) a high-abundance component corresponding to genuinely expressed genes. The resulting mixture density is shown in red. The decision boundary used to distinguish background from expressed genes corresponds to the intersection between the second and third components (vertical dashed line).

**Supplementary Table 1.** Complete list of the 672 Genes Of Interest (GOIs) selected for gene expression profiling of the hCMEC/D3 cell line. Gene symbols of 436 ion channels (i.e., the complete human channelome), 14 aquaporins, 90 ATPase pumps, 81 solute carriers specific for inorganic solutes, and 51 receptors (GPCRs and RTKs) are presented in aggregate, sorted alphabetically by column (next page). The same curated list is also available online, in CSV format, at the following GitHub address for programmatic browsing: <https://github.com/TCP-Lab/Endothelion/blob/main/data/in/ICT_set_v2.csv>.

**Supplementary Table 2. Solute carriers and pumps expressed in hCMEC/D3 cells**

| **Transporter/pump name** | **Gene** | **Function** | **Average mRNA TPMs** | **Location** | **Reference (s)** |
| --- | --- | --- | --- | --- | --- |
| F-type ATPase α subunit | *ATP5F1A* | Mitochondrial ATP synthase | 286.75 | Mitochondrial inner membrane | ([Lai et al., 2023](#_ENREF_69)) |
| F-type ATPase β subunit | *ATP5F1B* | Mitochondrial ATP synthase | 478.76 | Mitochondrial inner membrane | ([Lai et al., 2023](#_ENREF_69)) |
| F-type ATPase γ subunit | *ATP5F1C* | Mitochondrial ATP synthase | 228.80 | Mitochondrial inner membrane | ([Lai et al., 2023](#_ENREF_69)) |
| F-type ATPase δ subunit | *ATP5F1D* | Mitochondrial ATP synthase | 146.86 | Mitochondrial inner membrane | ([Lai et al., 2023](#_ENREF_69)) |
| F-type ATPase ε subunit | *ATP5F1E* | Mitochondrial ATP synthase | 78.72 | Mitochondrial inner membrane | ([Lai et al., 2023](#_ENREF_69)) |
| F-type ATPase B subunit | *ATP5PB* | Mitochondrial ATP synthase | 229.30 | Mitochondrial inner membrane | (Lai et al., 2023) |
| F-type ATPase C subunit | *ATP5MC1* | Mitochondrial ATP synthase | 191.71 | Mitochondrial inner membrane | (Lai et al., 2023) |
| F-type ATPase C subunit | *ATP5MC2* | Mitochondrial ATP synthase | 517.25 | Mitochondrial inner membrane | (Lai et al., 2023) |
| F-type ATPase C subunit | *ATP5MC3* | Mitochondrial ATP synthase | 224.14 | Mitochondrial inner membrane | (Lai et al., 2023) |
| F-type ATPase D subunit | *ATP5PD* | Mitochondrial ATP synthase | 342.06 | Mitochondrial inner membrane | (Lai et al., 2023) |
| F-type ATPase E subunit | *ATP5ME* | Mitochondrial ATP synthase | 369.96 | Mitochondrial inner membrane | (Lai et al., 2023) |
| F-type ATPase F2 subunit | *ATP5MF* | Mitochondrial ATP synthase | 921.26 | Mitochondrial inner membrane | (Lai et al., 2023) |
| F-type ATPase F6 subunit | *ATP5PF* | Mitochondrial ATP synthase | 243.11 | Mitochondrial inner membrane | (Lai et al., 2023) |
| ATP Synthase Membrane Subunit G | *ATP5MG* | Mitochondrial ATP synthase | 195.15 | Mitochondrial inner membrane | (Lai et al., 2023) |
| ATP Synthase Membrane Subunit J | *ATP5MJ* | Mitochondrial ATP synthase | 84.89 | Mitochondrial inner membrane | (Lai et al., 2023) |
| ATP Synthase Membrane Subunit K | *ATP5MK* | Mitochondrial ATP synthase | 551.26 | Mitochondrial inner membrane | (Lai et al., 2023) |
| ATP Synthase Peripheral Stalk Subunit OSCP | *ATP5PO* | Mitochondrial ATP synthase | 287.46 | Mitochondrial matrix | ([Ganapathi et al., 2022](#_ENREF_44)) |
| ATP Synthase Inhibitory Factor Subunit 1 | *ATP5IF1* | Natural inhibitor of the hydrolytic activity of the ATP synthase | 354.34 | Mitochondrial inner membrane | ([Mori et al., 2025](#_ENREF_98)) |
| Na^+^/K^+^ ATPase α-1 subunit | *ATP1A1* | 3 Na^+^ out : 2 K^+^ in for every ATP that is hydrolyzed | 242.25 | PM | ([Alexander et al., 2023b](#_ENREF_4)) |
| Na^+^/K^+^ ATPase β-1 subunit | *ATP1B1* | 3 Na^+^ out : 2 K^+^ in for every ATP that is hydrolyzed | 41.14 | PM | ([Alexander et al., 2023b](#_ENREF_4)) |
| Na^+^/K^+^ ATPase β-3 subunit | *ATP1B3* | 3 Na^+^ out : 2 K^+^ in for every ATP that is hydrolyzed | 166.64 | PM | ([Alexander et al., 2023b](#_ENREF_4)) |
| FXYD5 | *FXYD5* | Regulates Na^+^/K^+^ ATPase activity | 209.70 | PM | ([Miller and Davis, 2008](#_ENREF_90)) |
| SERCA1 | *ATP2A1* | 2 Ca^2+^ into the ER for every ATP that is hydrolyzed | 1.66 | ER | ([Alexander et al., 2023b](#_ENREF_4)) |
| SERCA2 | *ATP2A2* | 2 Ca^2+^ into the ER for every ATP that is hydrolyzed | 83.65 | ER | ([Alexander et al., 2023b](#_ENREF_4)) |
| PMCA1 | *ATP2B1* | 1 Ca^2+^ into the ER for every ATP that is hydrolyzed | 10.51 | PM | ([Alexander et al., 2023b](#_ENREF_4)) |
| PMCA4 | *ATP2B4* | 1 Ca^2+^ into the ER for every ATP that is hydrolyzed | 18.23 | PM | ([Alexander et al., 2023b](#_ENREF_4)) |
| SPCA1 | *ATP2C1* | 1 Ca^2+^, 1 Mn^2+^ into Golgi lumen for every ATP that is hydrolyzed | 55.39 | Golgi apparatus | ([Alexander et al., 2023b](#_ENREF_4)) |
| V-type ATPase V_0_ motor a1 subunit | *ATP6V0A1* | 3 to 4 H^+^ into lysosomal vesicles for every ATP that is hydrolyzed | 25.27 | Lysosomes | ([Finbow and Harrison, 1997](#_ENREF_42)) |
| V-type ATPase V_0_ motor a1 subunit | *ATP6V0A2* | 3 to 4 H^+^ into lysosomal vesicles for every ATP that is hydrolyzed | 12.79 | Lysosomes | ([Finbow and Harrison, 1997](#_ENREF_42)) |
| V-type ATPase V_0_ motor a3 subunit | *TCIRG1* | 3 to 4 H^+^ into lysosomal vesicles for every ATP that is hydrolyzed | 74.65 | Lysosomes | ([Finbow and Harrison, 1997](#_ENREF_42)) |
| V-type ATPase V_0_ motor b subunit | *atp6v0b* | 3 to 4 H^+^ into lysosomal vesicles for every ATP that is hydrolyzed | 115.74 | Lysosomes | ([Finbow and Harrison, 1997](#_ENREF_42)) |
| V-type ATPase V_0_ motor c subunit | *ATP6V0C* | 3 to 4 H^+^ into lysosomal vesicles for every ATP that is hydrolyzed | 234.00 | Lysosomes | [Finbow and Harrison, 1997](#_ENREF_32)) |
| V-type ATPase V_0_ motor d1 subunit | *ATP6V0D1* | 3 to 4 H^+^ into lysosomal vesicles for every ATP that is hydrolyzed | 85.04 | Lysosomes | ([Finbow and Harrison, 1997](#_ENREF_42)) |
| V-type ATPase V_0_ motor e1 subunit | *ATP6V0E1* | 3 to 4 H^+^ into lysosomal vesicles for every ATP that is hydrolyzed | 150.40 | Lysosomes | ([Finbow and Harrison, 1997](#_ENREF_42)) |
| V-type ATPase V_0_ motor e2 subunit | *ATP6V0E2* | 3 to 4 H^+^ into lysosomal vesicles for every ATP that is hydrolyzed | 30.16 | Lysosomes | ([Finbow and Harrison, 1997](#_ENREF_42)) |
| V-type ATPase V_1_ motor A subunit | *ATP6V1A* | 3 to 4 H^+^ into lysosomal vesicles for every ATP that is hydrolyzed | 29.25 | Lysosomes | ([Finbow and Harrison, 1997](#_ENREF_42)) |
| V-type ATPase V_1_ motor B2 subunit | *ATP6V1B2* | 3 to 4 H^+^ into lysosomal vesicles for every ATP that is hydrolyzed | 49.29 | Lysosomes | ([Finbow and Harrison, 1997](#_ENREF_42)) |
| V-type ATPase V_1_ motor C1 subunit | *ATP6V1C1* | 3 to 4 H^+^ into lysosomal vesicles for every ATP that is hydrolyzed | 33.16 | Lysosomes | ([Finbow and Harrison, 1997](#_ENREF_42)) |
| V-type ATPase E_1_ motor D subunit | *ATP6V1D* | 3 to 4 H^+^ into lysosomal vesicles for every ATP that is hydrolyzed | 82.83 | Lysosomes | ([Finbow and Harrison, 1997](#_ENREF_42)) |
| V-type ATPase E_1_ motor E1 subunit | *ATP6V1E1* | 3 to 4 H^+^ into lysosomal vesicles for every ATP that is hydrolyzed | 136.04 | Lysosomes | ([Finbow and Harrison, 1997](#_ENREF_42)) |
| V-type ATPase E_1_ motor E1 subunit | *ATP6V1E2* | 3 to 4 H^+^ into lysosomal vesicles for every ATP that is hydrolyzed | 2.94 | Lysosomes | ([Finbow and Harrison, 1997](#_ENREF_42)) |
| V-type ATPase V_1_ motor F subunit | *ATP6V1F* | 3 to 4 H^+^ into lysosomal vesicles for every ATP that is hydrolyzed | 215.76 | Lysosomes | ([Finbow and Harrison, 1997](#_ENREF_42)) |
| V-type ATPase V_1_ motor G1 subunit | *ATP6V1G1* | 3 to 4 H^+^ into lysosomal vesicles for every ATP that is hydrolyzed | 72.48 | Lysosomes | ([Finbow and Harrison, 1997](#_ENREF_42)) |
| V-type ATPase V_1_ motor H subunit | *ATP6V1H* | 3 to 4 H^+^ into lysosomal vesicles for every ATP that is hydrolyzed | 46.91 | Lysosomes | ([Finbow and Harrison, 1997](#_ENREF_42)) |
| Cu^+^-ATPase 1 | *ATP7A* | Transports Cu^+^ inside the cytosol (PM) or into the TGN lumen (unknown stoichiometry) | 4.52 | PM and TGN | ([Lutsenko et al., 2007](#_ENREF_84)) |
| Cu^+^-ATPase 2 | *ATP7B* | Transports Cu^+^ inside the cytosol (PM) or into the TGB lumen (unknown stoichiometry) | 1.38 | PM and TGN | ([Lutsenko et al., 2007](#_ENREF_84)) |
| ATP8B1 | *ATP8B1* | Translocates PC from one side of the phospholipid bilayer to the other | 34.62 | PM | ([Shin and Takatsu, 2019](#_ENREF_124)) |
| ATP8B2 | *ATP8B2* | PC | 21.55 | PM | ([Shin and Takatsu, 2019](#_ENREF_124)) |
| ATP8B3 | *ATP8B3* | PS | 4.56 | N.D. | ([Shin and Takatsu, 2019](#_ENREF_124)) |
| ATP9A | *ATP9A* | N.D. | 4.36 | EE, RE, and Golgi | ([Shin and Takatsu, 2019](#_ENREF_124)) |
| ATP9B | *ATP9B* | N.D. | 8.13 | Golgi | ([Shin and Takatsu, 2019](#_ENREF_124)) |
| ATP10D | *ATP10D* | N.D. | 18.73 | PM | ([Shin and Takatsu, 2019](#_ENREF_124)) |
| ATP11A | *ATP11A* | PS and PE | 17.39 | PM | ([Shin and Takatsu, 2019](#_ENREF_124)) |
| ATP11B | *ATP11B* | PS > PE | 31.26 | EE and RE | ([Shin and Takatsu, 2019](#_ENREF_124)) |
| ATP11C | *ATP11C* | PS > PE | 16.98 | PM | ([Shin and Takatsu, 2019](#_ENREF_124)) |
| ATPase 13A1 | *ATP13A1* | ATP-dependent extraction of mislocalized mitochondrial transmembrane proteins | 32.28 | ER | ([McKenna et al., 2020](#_ENREF_89)) |
| ATPase cation transporting 13A2 | *ATP13A2* | Lysosomal polyamine exporter with high affinity for spermine | 32.43 | Lysosomes | ([van Veen et al., 2020](#_ENREF_132)) |
| ATPase 13A3 | *ATP13A3* | Component of the mammalian polyamine transport system | 52.45 | Endosomes | ([Alexander et al., 2023b](#_ENREF_4)) |
| NCX1 | *SLC8A1* | 3 Na^+^ (in) : 1 Ca^2+^ (out) | 1.39 | PM | (Alexander et al., 2023b) |
| NKCX6 | *SLC8B1* | 4Na^+^: (1Ca^2+^ + 1K^+^) | 20.48 | Mitochondrial inner membrane | (Alexander et al., 2023b) |
| NHE1 | *SLC9A1* | 1 Na^+^ (in) : 1 H^+^ (out) | 22.17 | PM | (Alexander et al., 2023b) |
| NHE3 | *SLC9A3* | 1 Na^+^ (in) : 1 H^+^ (out) | 1.86 | PM | (Alexander et al., 2023b) |
| NHE5 | *SLC9A5* | 1 Na^+^ (in) : 1 H^+^ (out) | 2.05 | PM | (Alexander et al., 2023b) |
| NHE6 | *SLC9A6* | 1 Na^+^ (in) : 1 H^+^ (out) (referred to endosomal lumen) | 10.92 | EE and RE | (Alexander et al., 2023b) |
| NHE7 | *SLC9A7* | 1 Na^+^ (in) : 1 H^+^ (out) (referred to Golgi lumen) | 6.17 | TGN | (Alexander et al., 2023b) |
| NHE8 | *SLC9A8* | 1 Na^+^ (in) : 1 H^+^ (out) | 5.09 | PM | (Alexander et al., 2023b) |
| NHA2 | *SLC9B2* | 1 Na^+^ (in) : 1 H^+^ (out) | 8.68 | Mitochondrial inner membrane | (Alexander et al., 2023b) |
| NKCC1 | *SLC12A2* | 1 Na^+^ : 1 K^+^ : 2 Cl^-^ (in) | 10.44 | PM | (Alexander et al., 2023b) |
| PiT1 | *SLC20A1* | > 1 Na^+^ : 1 HPO_4_^2-^ (in) | 83.23 | PM | (Alexander et al., 2023b) |
| PiT2 | *SLC20A2* | > 1 Na^+^ : 1 HPO_4_^2-^ (in) | 28.24 | PM | (Alexander et al., 2023b) |
| NKCX1 | *SLC24A1* | 4Na^+^:(1Ca^2+^ + 1K^+^) | 5.50 | PM | (Alexander et al., 2023b) |
| UCP5 | *SLC25A14* | H^+^ (in) | 13.55 | Mitochondrial inner membrane | (Alexander et al., 2023b) |
| Mitoferrin-2 | *SLC25A28* | Iron (in) | 43.08 | Mitochondrial inner membrane | ([Paradkar et al., 2009](#_ENREF_107)) |
| Mitoferrin-1 | *SLC25A37* | Iron (in) | 38.20 | Mitochondrial inner membrane | ([Chen et al., 2009](#_ENREF_32)) |
| ZnT1 | *SLC30A1* | Zn^2+^ (in) : H^+^ (out) | 15.92 | PM | ([Kambe et al., 2015](#_ENREF_58)) |
| ZnT4 | *SLC30A4* | Zn^2+^ (out) : H^+^ (in) | 3.18 | Endosomes, lysosomes, TGN, Golgi apparatus | (Kambe et al., 2015) |
| ZnT5 | *SLC30A5* | Zn^2+^ (out) : H^+^ (in) | 33.31 | Golgi apparatus | (Kambe et al., 2015) |
| ZntT6 | *SLC30A6* | Zn^2+^ (out) : H^+^ (in) | 14.49 | TGN | (Kambe et al., 2015) |
| ZnT7 | *SLC30A7* | Zn^2+^ (out) : H^+^ (in) | 7.42 | TGN | (Kambe et al., 2015) |
| ZnT9 | *SLC30A9* | Zn^2+^ (in) : H^+^ (out) | 27.37 | PM | (Alexander et al., 2023b) |
| CTR1 | *SLC31A1* | Cu^2+^ (in) | 19.98 | PM | (Alexander et al., 2023b) |
| CTR2 | *SLC31A2* | Cu^2+^ (in) | 4.70 | Vacuoles/Vesicles | (Alexander et al., 2023b) |
| ZIP1 | *SLC39A1* | Zn^2+^ (in) (unknown mechanism) | 88.92 | PM | (Kambe et al., 2015) |
| ZIP3 | *SLC39A3* | Zn^2+^ (in) (unknown mechanism) | 45.38 | PM | (Kambe et al., 2015) |
| ZIP4 | *SLC39A4* | Zn^2+^ (in) (unknown mechanism) | 16.25 | PM | (Kambe et al., 2015) |
| ZIP6 | *SLC39A6* | Zn^2+^ (in) (unknown mechanism) | 36.02 | PM | (Kambe et al., 2015) |
| ZIP7 | *SLC39A7* | Zn^2+^ (in) (unknown mechanism) | 110.58 | ER | (Kambe et al., 2015) |
| ZIP10 | *SLC39A10* | Zn^2+^ (in) (unknown mechanism) | 10.49 | PM | ([Kambe et al., 2015](#_ENREF_58)) |
| ZIP13 | *SLC39A13* | Zn^2+^ (in) (unknown mechanism) | 67.99 | Golgi apparatus | (Kambe et al., 2015) |
| ZIP14 | *SLC39A14* | Zn^2+^ (in) (unknown mechanism) | 33.86 | PM | (Kambe et al., 2015) |
| MgtE | *SLC41A1* | Na^+^ (in) : Mg^2+^ (out) (unknown stoichiometry) | 20.39 | PM | ([Kolisek et al., 2012](#_ENREF_66)) |
| Solute carrier family 41 member 2 | *SLC41A2* | Na^+^ (in) : Mg^2+^ (out) (unknown stoichiometry) | 6.60 | PM | ([Alexander et al., 2023b](#_ENREF_4)) |
| Magnesium transporter 1 | *MAGT1* | Mg^2+^ | 48.50 | PM | (Alexander et al., 2023b) |
| Tumor suppressor candidate 3 | *TUSC3* | Mg^2+^ | 47.65 | PM | (Alexander et al., 2023b) |
| AE | *SLC4A2* | 1 Cl^-^ (in) : 1 HCO_3_^-^ (out) | 87.73 | PM | (Alexander et al., 2023b) |
| NBCn1 | *SLC4A7* | 1 Na^+^ : 1 HCO_3_^-^ (out) or 1 Na^+^ : CO_3_^2*^ | 12.16 | PM | (Alexander et al., 2023b) |
| NDCBE | *SLC4A8* | 1 Na^+^ : 2HCO_3_^-^ (in) : 1 Cl^-^ (out) | 2.33 | PM | Alexander et al., 2023b) |
| BTR1 | *SLC4A11* | Cl^-^, NaHCO_3_  (unknown stoichiometry) | 6.50 | PM | Alexander et al., 2023b) |
| RFVT2 | *SLC52A2* | Riboflavin (in) | 54.99 | PM | Alexander et al., 2023b) |
| XPR1 | *XPR1* | Phosphate (out) | 17.40 | PM | Alexander et al., 2023b) |

Abbreviations: ATP8B1: phospholipid-transporting ATPase IC; ATP8B2: Phospholipid-transporting ATPase ID; ATP8B3: phospholipid-transporting ATPase IK; ATP9A: probable phospholipid-transporting ATPase IIA; ATP9B: probable phospholipid-transporting ATPase IIB; ATP10D: probable phospholipid-transporting ATPase VD; ATP11A: probable phospholipid-transporting ATPase IH; ATP11B: probable phospholipid-transporting ATPase IF; ATP11C: phospholipid-transporting ATPase IG; CTR: Cu^2+^ transporter; EE: early endosomes; FXYD5: FXYD Domain Containing Ion Transport Regulator 5; NCX1: Na^+^/Ca^2+^ exchanger 1; NKCX: Sodium/potassium/calcium exchanger; N.D.: not determined; NHE: Na^+^/H^+^ exchanger; NKCC: basolateral Na-K-Cl symporter; PC: phosphatidylcholine; PE: phosphatidylethanolamine; PS: phosphatidylserine; PiT: sodium-dependent phosphate transporter ; RE: recycling endosomes; UCP: uncoupling protein; TGN: Trans-Golgi Network; ZIP: Zrt-, Irt-related proteins; ZnT: Zn^2+^ transporter.

**Supplementary Table 3. Ion channels and aquaporins expressed in hCMEC/D3 cells**

| **Channel name** | **Gene** | **Selectivity** | **mRNA average counts** | **Functional role in hCMEC/D3 cells** | **Location and Endogenous activators** | **Reference (s)** |
| --- | --- | --- | --- | --- | --- | --- |
| nAChR α5 subunit | *CHRNA5* | Na^+^, K^+^, and Ca^2+^ | 7.23 | N.D. | PM; acetylcholine | ([Moccia et al., 2004](#_ENREF_96); [Alexander et al., 2023c](#_ENREF_5)) |
| Dupα7  Human variant of nAchR α7 | *CHRFAM7A* | Binds to nAchR7 | 1.29 | N.D. | PM; acetylcholine (negative dominant of nAchR7) | ([Dang et al., 2015](#_ENREF_33)) |
| nAChR β1 subunit | *CHNRB1* | Na^+^, K^+^, and Ca^2+^ | 28.27 | N.D. | PM; acetylcholine | ([Alexander et al., 2023c](#_ENREF_5)) |
| P2X4 receptor | *P2RX4* | Na^+^, K^+^, and Ca^2+^ | 17.60 | Expressed in hCMEC/D3 cells, unknown function | PM; ATP | ([Bintig et al., 2012](#_ENREF_19)) |
| P2X5 receptor | *P2RX5* | Na^+^, K^+^, and Ca^2+^ | 4.14 | Expressed in hCMEC/D3 cells, unknown function | PM; ATP | ([Bintig et al., 2012](#_ENREF_19)) |
| P2X7 receptor | *P2RX7* | Na^+^, K^+^, and Ca^2+^ | 1.38 | Expressed in hCMEC/D3 cells, unknown function | PM; ATP | ([Bintig et al., 2012](#_ENREF_17)) |
| GABA_A_R ε subunit | *GABRE* | Cl^-^ | 15.19 | N.D. | PM; GABA | ([Alexander et al., 2023c](#_ENREF_5)) |
| GlyR β subunit | *GLRB* | Cl^-^ | 7.29 | Angiogenesis** | PM; glycine | ([Alexander et al., 2023c](#_ENREF_5); [Xu et al., 2024](#_ENREF_139)) |
| AQP3 | *AQP3* | H_2_O and H_2_O_2_ | 1.52 | N.D. | PM; Constitutively open | ([Alexander et al., 2023c](#_ENREF_5)) |
| AQP5 | *AQP5* | H_2_O and H_2_O_2_ | 1.03 | N.D. | PM; Constitutively open | ([Alexander et al., 2023c](#_ENREF_5)) |
| K_V_4.1 | *KCND1* | K^+^ | 1.38 | N.D. | PM; membrane depolarization | ([Alexander et al., 2023c](#_ENREF_5)) |
| K_IR_2.1 | *KCNJ2* | K^+^ | 2.08 | Detection of neuronal activity | PM; a modest increase (up to 10 mM) in extracellular K^+^ concentration | ([Longden et al., 2017](#_ENREF_80)) |
| TREK1  (K2p2.1) | *KCNK2* | K^+^ | 1.40 | Leukocyte trafficking** | PM; Background current, arachidonic acid, extracellular acidification, membrane stretch, temperature (17-40°C) | ([Bittner et al., 2013](#_ENREF_20); [Avalos Prado et al., 2022](#_ENREF_10)) |
| TWIK-2 (K2p6.1) | *KCNK6* | K^+^ | 3.32 | N.D. | PM and ELs; Background current and arachidonic acid | ([Lloyd et al., 2009](#_ENREF_79); [Bobak et al., 2017](#_ENREF_21)) |
| IK_Ca_  (K_Ca_3.1) | *KCNN4* | K^+^ | 3.80 | Hyperpolarization* and CBF increase** | PM; Increase in [Ca^2+^]_i_ | ([Earley, 2011](#_ENREF_40); [Berra-Romani et al., 2023](#_ENREF_14)) |
| BK_Ca_  (K_Ca_1.1) | *KCNMA1* | K^+^ | 8.35 | N.D. | PM; Membrane depolarization and increase in [Ca^2+^]_i_ | ([Alexander et al., 2023c](#_ENREF_5)) |
| K_Na_1.2  (Slo2.1) | *KCNT2* | K^+^ | 1.55 | N.D. | PM; Intracellular Na^+^ and Cl^-^, cell swelling | ([Tejada et al., 2014](#_ENREF_127); [Alexander et al., 2023c](#_ENREF_5)) |
| VRAC (Swell1) | *LRRC8A* | Cl^-^ | 61.25 | BBB integrity?** | PM; Cell swelling | ([Karakas et al., 2025](#_ENREF_61); [Tian et al., 2025](#_ENREF_129)) |
| VRAC (Swell1) | *LRRC8C* | Cl^-^ | 10.79 | BBB integrity? | PM; Must assemble with LRRC8A | ([Kern et al., 2023](#_ENREF_63); [Karakas et al., 2025](#_ENREF_61)) |
| VRAC (Swell1) | *LRRC8D* | Cl^-^ | 19.66 | BBB integrity? | PM; Cell swelling | ([Karakas et al., 2025](#_ENREF_61); [Quinodoz et al., 2025](#_ENREF_113)) |
| VRAC (Swell1) | *LRRC8E* | Cl^-^ | 9.57 | BBB integrity? | PM; Must assemble with LRRC8A | ([Bertelli et al., 2021](#_ENREF_18); [Karakas et al., 2025](#_ENREF_61)) |
| CaCC (Bestrophin 1) | *BEST1* | Cl^-^ | 1.59 | N.D. | PM; Intracellular Ca^2+^ | ([Owji et al., 2021](#_ENREF_105)) |
| CaCC (TMEM16F) | *ANO6* | Cl^-^ and phospholipids | 30.96 | Clot formation? | PM; Intracellular Ca^2+^ (low sensitivity) | ([Schreiber et al., 2018](#_ENREF_121); [Bonson et al., 2025](#_ENREF_23); [Kageyama et al., 2025](#_ENREF_57)) |
| CaCC (TMEM16G) | *ANO7* | Phospholipids, Cl^-^ | 2.06 | N.D. | PM; Intracellular Ca^2+^ | ([Duran et al., 2012](#_ENREF_39); [Kunzelmann et al., 2024](#_ENREF_68)) |
| CaCC  (TMEM16H) | *ANO8* | Phospholipids, Cl^-^ | 4.69 | N.D. | ER-PM junctions (enriched in the ER); | ([Kunzelmann et al., 2024](#_ENREF_68)) |
| CaCC  (TMEM16K) | *ANO10* | Phospholipids, Cl^-^? | 31.41 | N.D. | ER; Intracellular Ca^2+^ | ([Bushell et al., 2019](#_ENREF_27); [Le and Yang, 2021](#_ENREF_70)) |
| ClC-2 | *CLCN2* | Cl^-^ | 4.51 | N.D. | PM; Membrane hyperpolarization, cell swelling, weak extracellular acidification | ([Wang et al., 2017](#_ENREF_135); [Alexander et al., 2023c](#_ENREF_5)) |
| ClC-3 | *CLCN3* | 2Cl^-^/H^+^ antiporter | 16.27 | N.D. | EL vesicles; Constitutive activity, possibly enhanced by CaMKII | ([Jentsch and Pusch, 2018](#_ENREF_55); [Alexander et al., 2023c](#_ENREF_5)) |
| ClC-4 | *CLCN4* | 2Cl^-^/H^+^ antiporter | 1.98 | N.D. | Possibly located in EL vesicles; Activated by positive voltages | ([Picollo and Pusch, 2005](#_ENREF_112); [Jentsch and Pusch, 2018](#_ENREF_55)) |
| ClC-5 | *CLCN5* | 2Cl^-^/H^+^ antiporter | 2.68 | N.D. | EL vesicles; Activated by positive voltages (>20 mV) | ([Jentsch and Pusch, 2018](#_ENREF_55)) |
| ClC-6 | *CLCN6* | 2Cl^-^/H^+^ antiporter | 12.00 | N.D. | Late endosomes; Activated by positive voltages | ([Neagoe et al., 2010](#_ENREF_100); [Jentsch and Pusch, 2018](#_ENREF_55)) |
| ClC-7 | *CLCN7* | 2Cl^-^/H^+^ antiporter | 58.92 | N.D. | Lysosomes; Activated by positive voltages (>20 mV) | ([Jentsch and Pusch, 2018](#_ENREF_55); [Alexander et al., 2023c](#_ENREF_5)) |
| CLIC1 | *CLIC1* | Cl^-^ | 514.95 | BBB integrity?** | Cytoplasm, PM, mitochondria, nucleoplasm, intracellular membranes; Zn^2+^-induced membrane insertion, activated by low pH | ([Gururaja Rao et al., 2018](#_ENREF_47); [Varela et al., 2022](#_ENREF_133); [Xiang et al., 2025](#_ENREF_138)) |
| CLIC3 | *CLIC3* | Cl^-^ | 14.12 | N.D. | PM, nucleus and MERCS | ([Raut et al., 2024](#_ENREF_114); [Sanghvi et al., 2025](#_ENREF_120)) |
| CLIC4 | *CLIC4* | Cl^-^ | 271.54 | Brain angiogenesis**, pyroptosis** | PM, cytoplasm, nucleus, Golgi apparatus and MERCS;  Redox-sensitive | ([Littler et al., 2005](#_ENREF_76); [Lucitti et al., 2015](#_ENREF_81); [Gururaja Rao et al., 2018](#_ENREF_47); [Zhang et al., 2024c](#_ENREF_146); [Sanghvi et al., 2025](#_ENREF_120)) |
| CLNS1A (pICln) | *CLNS1A* | Cl^-^ | 131.47 | N.D. | PM; Volume-sensitive but no longer regarded as a VRAC | ([Furst et al., 2006](#_ENREF_43); [Alexander et al., 2023c](#_ENREF_5)) |
| PACC1 | *PACC1* | Cl^-^ | 9.44 | ND. | PM; Extracellular acidification | ([Ruan et al., 2020](#_ENREF_116)) |
| Na_v_1.5 | *SCN5A* | Na^+^ | 1.83 | N.D. | PM; Membrane depolarization | ([Alexander et al., 2023c](#_ENREF_5)) |
| Na_v_1.6 | *SCN8A* | Na^+^ | 2.53 | N.D. | PM; Membrane depolarization | ([Alexander et al., 2023c](#_ENREF_5)) |
| Na_v_1.7 | *SCN9A* | Na^+^ | 1.18 | N.D. | PM; Membrane depolarization | ([Alexander et al., 2023c](#_ENREF_5)) |
| Na_Vi_ | *NALCN* | Na^+^ | 2.49 |  | PM; Constitutively open, but also activated downstream GqPCRs in an SFK-dependent manner | ([Monteil et al., 2024](#_ENREF_97)) |
| α-ENaC | *SCNN1A* | Na^+^ | 6.02 | BBB integrity** | PM; Constitutively open, sensitive to shear stress | ([Sternak et al., 2018](#_ENREF_125); [Zhang et al., 2022](#_ENREF_143)) |
| δ-ENaC | *SCNN1D* | Na^+^ | 2.95 | N.D. | PM; Constitutively open, less sensitive to shear stress | ([Paudel et al., 2021](#_ENREF_109)) |
| HCN2 | *HCN2* | Na^+^ and K^+^ | 4.37 | N.D. | PM; Membrane hyperpolarization, cyclic AMP>cyclic GMP | ([Alexander et al., 2023c](#_ENREF_5)) |
| HCN3 | *HCN3* | Na^+^ and K^+^ | 4.21 | N.D. | PM; Membrane hyperpolarization | ([Alexander et al., 2023c](#_ENREF_5)) |
| H_v_1 | *HVCN1* | H^+^ | 2.53 | N.D. | PM; Membrane depolarization and sensitive to the transmembrane pH gradient | ([DeCoursey, 2018](#_ENREF_35)) |
| ASIC1 | *ASIC1* | Na^+^, K^+^, and Ca^2+^ | 4.41 | Vasodilation?** | PM; Extracellular acidification | ([Lin et al., 2014](#_ENREF_74); [Zhang et al., 2020](#_ENREF_144)) |
| ASIC3 | *ASIC3* | Na^+^, K^+^, and Ca^2+^ | 1.83 | N.D. | PM; Extracellular acidification | ([Delaunay et al., 2012](#_ENREF_36); [Zhang et al., 2020](#_ENREF_144)) |
| CALHM2 | *CALHM2* | Cations, anions, ATP | 27.97 | N.D. | PM; Membrane depolarization and extracellular Ca^2+^ | ([Ma et al., 2016](#_ENREF_86); [Liu et al., 2020](#_ENREF_77)) |
| CALHM5 | *CALHM5* | Cations, anions, ATP | 1.48 | N.D. | PM; Membrane depolarization and extracellular Ca^2+^ | ([Ma et al., 2016](#_ENREF_86); [Liu et al., 2020](#_ENREF_77)) |
| Piezo1 | *PIEZO1* | Na^+^, K^+^, Ca^2+^ and Mg^2+^ | 103.83 | Shear stress-dependent Ca^2+^ entry and CBF modulation | PM; Mechano-sensitive (e.g. shear stress) | ([Harraz et al., 2022](#_ENREF_48); [Lim et al., 2024](#_ENREF_73)) |
| TMC-7 | *TMC7* | ? | 1.22 | N.D. | PM; Inhibits Piezo2 channel activity | ([Zhang et al., 2024b](#_ENREF_145)) |
| TACAN | *TMEM120A* | Na^+^, K^+^ and Ca^2+^ | 26.03 | N.D. | PM; Mechano-sensitive | ([Kang and Lee, 2024](#_ENREF_60)) |
| TMEM63A | *TMEM63A* | Na^+^, K^+^ and Ca^2+^ | 18.56 | N.D. | Lysosome; Mechano-sensitive | ([Zheng et al., 2023](#_ENREF_148); [Liu et al., 2025](#_ENREF_78)) |
| TMEM63B | *TMEM63B* | Na^+^, K^+^ and Ca^2+^ | 21.72 | N.D. | PM and Lysosomes; Mechanosensitive | ([Zheng et al., 2023](#_ENREF_148); [Chen et al., 2024](#_ENREF_29)) |
| TRPC1 | *TRPC1* | Na^+^, K^+^ and Ca^2+^ | 7.24 | BBB permeability** | PM; Activated by the depletion of the ER Ca^2+^ store or by membrane stretch | ([Berrout et al., 2012](#_ENREF_17); [Moccia et al., 2023a](#_ENREF_93)) |
| TRPC4 | *TRPC4* | Na^+^, K^+^ and Ca^2+^ | 1.54 | BBB permeability** | PM; Activated by the depletion of the ER Ca^2+^ store | ([Balbuena et al., 2012](#_ENREF_13); [Moccia et al., 2023a](#_ENREF_93); [Dragoni et al., 2025](#_ENREF_38)) |
| TRPM2 | *TRPM2* | Na^+^, K^+^ and Ca^2+^ | 5.25 | BBB permeability** | PM; Oxidative stress | ([Park et al., 2014](#_ENREF_108); [Ding et al., 2021](#_ENREF_37); [Zong et al., 2024](#_ENREF_150)) |
| TRPM4 | *TRPM4* | Na^+^ and K^+^ | 13.81 | BBB permeability** and volume regulation** | PM; Intracellular Ca^2+^ | ([Negri et al., 2019](#_ENREF_101); [Ma et al., 2023](#_ENREF_85); [Alquisiras-Burgos et al., 2024](#_ENREF_6)) |
| TRPM7 | *TRPM7* | K^+^, Ca^2+^ and Mg^2+^ | 17.58 | BBB permeability** | PM; A reduction in intracellular Mg^2+^ and Mg-nucleotides | ([Zhu et al., 2018](#_ENREF_149); [Negri et al., 2019](#_ENREF_101)) |
| TRPV1 | *TRPV1* | Na^+^, K^+^ and Ca^2+^ | 4.05 | Expressed in hCMEC/D3 cells, unknown function | PM; Temperature >41°C, oxidative stress, endocannabinoids | ([Luo et al., 2020](#_ENREF_83); [Negri et al., 2020b](#_ENREF_103)) |
| TRPV2 | *TRPV2* | Na^+^, K^+^ and Ca^2+^ | 25.06 | Angiogenesis and BBB integrity* | PM; Temperature >52°C, oxidative stress, endocannabinoids | ([Luo et al., 2019](#_ENREF_82); [Luo et al., 2020](#_ENREF_83)) |
| TRPV4 | *TRPV4* | Na^+^, K^+^ and Ca^2+^ | 2.77 | NO release* and NVC ** | PM; PIP_2_ hydrolysis and arachidonic acid | ([Harraz et al., 2018](#_ENREF_49); [Berra-Romani et al., 2019](#_ENREF_15); [Moccia and Dragoni, 2025](#_ENREF_95)) |
| TRPML1 | *MCOLN1* | Na^+^, K^+^, Ca^2+^ and Fe^2+^ | 27.86 | NO release* | endosome/lysosome; ROS and PI(3,5)P_2_ | ([Brunetti et al., 2024](#_ENREF_24)) |
| Polycystin-1 | *PKD1* | Adhesion or mechanosensory reception | 49.08 | N.D. | Primary cilium | ([Boletta and Caplan, 2025](#_ENREF_22)) |
| Polycystin-2  (TRPP2) | *PKD2* | Na^+^, K^+^ and Ca^2+^ | 25.95 | BBB permeability** | Primary cilium; Mechanical stimuli | ([Berrout et al., 2012](#_ENREF_17); [Alexander et al., 2023c](#_ENREF_5); [Thirugnanam et al., 2023](#_ENREF_128)) |
| Orai1 | *ORAI1* | Ca^2+^ | 31.27 | NO release * | PM; Activated by depletion of the ER Ca^2+^ store | ([Zuccolo et al., 2019b](#_ENREF_152); [Negri et al., 2020a](#_ENREF_102); [Negri et al., 2022](#_ENREF_104); [Moccia et al., 2023a](#_ENREF_93)) |
| Orai2 | *ORAI2* | Ca^2+^ | 9.48 | Expressed in hCMEC/D3 cells | PM; Dominant negative of Orai1 | ([Zuccolo et al., 2019b](#_ENREF_152); [Yoast et al., 2020](#_ENREF_141)) |
| Orai3 | *ORAI3* | Ca^2+^ | 17.76 | Expressed in hCMEC/D3 cells | PM; Dominant negative of Orai1** | [Zuccolo et al., 2019](#_ENREF_65); [Yoast et al., 2020](#_ENREF_58)) |
| CatSper1 | *CATSPER1* | Ca^2+^ | 2.75 | N.D. | Flagellum in the sperm; Constitutively active, potentiated by intracellular alkalinization and reproductive steroids and prostaglandins | ([Kirichok et al., 2006](#_ENREF_65); [Jeschke et al., 2021](#_ENREF_56); [Alexander et al., 2023c](#_ENREF_5)) |
| CatSper2 | *CATSPER2* | Ca^2+^ | 2.31 | N.D. | Flagellum in the sperm; Constitutively active, potentiated by intracellular alkalinization and reproductive steroids and prostaglandins | ([Alexander et al., 2023c](#_ENREF_5)) |
| InsP_3_R1 | *ITPR1* | Ca^2+^ | 6.63 | ER Ca^2+^ release and NO release** | ER; InsP_3_ and Ca^2+^ | ([Zuccolo et al., 2017](#_ENREF_153); [Zuccolo et al., 2019a](#_ENREF_151); [Moccia et al., 2023b](#_ENREF_94)) |
| InsP_3_R2 | *ITPR2* | Ca^2+^ | 12.36 | ER Ca^2+^ release | ER; InsP_3_ and Ca^2+^ | ([Zuccolo et al., 2017](#_ENREF_153); [Zuccolo et al., 2019a](#_ENREF_151); [Moccia et al., 2023b](#_ENREF_94)) |
| InsP_3_R3 | *ITPR3* | Ca^2+^ | 36.22 | ER Ca^2+^ release* | ER; InsP_3_ and Ca^2+^ | ([Zuccolo et al., 2019b](#_ENREF_152); [Negri et al., 2020a](#_ENREF_102); [Negri et al., 2022](#_ENREF_104); [Moccia et al., 2023b](#_ENREF_94)) |
| TPC1 | *TPCN1* | Ca^2+^ | 19.96 | EL Ca^2+^ release* | EL; Ca^2+^ release | ([Zuccolo et al., 2019b](#_ENREF_152); [Berra-Romani et al., 2020](#_ENREF_16); [Negri et al., 2020a](#_ENREF_102); [Negri et al., 2022](#_ENREF_104); [Moccia et al., 2023b](#_ENREF_94)) |
| TPC2 | *TPCN2* | Ca^2+^ | 5.55 | EL Ca^2+^ release* | EL; Ca^2+^ release | ([Zuccolo et al., 2019b](#_ENREF_152); [Negri et al., 2020a](#_ENREF_102); [Negri et al., 2022](#_ENREF_104); [Moccia et al., 2023b](#_ENREF_94)) |
| TMBIM1 | *TMBMI1* | Ca^2+^ | 93.60 | N.D. | Endosomes and lysosomes; Ca^2+^ leakage | ([Lisak et al., 2015](#_ENREF_75)) |
| TMBIM2 (FAIM2) | *FAIM2* | Ca2+ | 1.63 | N.D. | ER and Golgi; Ca2+ leakage | (Lisak et al., 2015) |
| TMBIM4 | *TMBIM4* | Ca^2+^ | 74.81 | N.D. | ER and Golgi; Ca^2+^ leakage | ([Lisak et al., 2015](#_ENREF_75)) |
| TMBIM6 | *TMBIM6* | Ca^2+^ | 439.61 | N.D. | ER; Ca^2+^ leakage | ([Kim et al., 2008](#_ENREF_64); [Bultynck et al., 2012](#_ENREF_26)) |
| Mitsugumin 23 | *TMEM109* | Ca^2+^ | 106.56 | N.D. | ER; Ca^2+^ leakage | PMID: 9720923; PMID: 21381722 |
| TMCO1 | *TMCO1* | Ca^2+^ | 111.26 | N.D. | ER; Ca^2+^ load-activated Ca^2+^ channel | ([Wang et al., 2016](#_ENREF_136)) |
| Presenilin 1 | *PSEN1* | Ca^2+^ | 45.66 | N.D. | ER; Ca^2+^ leakage | ([Tu et al., 2006](#_ENREF_131)) |
| TRIC-A | *TMEM38A* | K^+^ | 3.46 | N.D. | ER; K^+^ efflux counteracting Ca^2+^ release | ([Venturi et al., 2013](#_ENREF_134)) |
| TRIC-B | *TMEM38B* | K^+^ | 7.92 | N.D. | ER; K^+^ efflux counteracting Ca^2+^ release | ([Venturi et al., 2013](#_ENREF_134)) |
| TMEM175 | *TMEM175* | K^+^ | 12.05 | N.D. | Lysosomes | ([Pergel et al., 2021](#_ENREF_110)) |
| CLCC1 | *CLCC1* | Cl^-^ | 21.16 | N.D. | ER; Cl^-^ influx counteracting Ca^2+^ release | ([Guo et al., 2023](#_ENREF_46)) |
| MCU | *MCU* | Ca^2+^ | 22.60 | N.D. | Mitochondrial inner membrane; mitochondrial Ca^2+^ influx | ([Alevriadou et al., 2017](#_ENREF_2)) |
| MICU1 | *MICU1* | Controls MCU activity | 47.75 | N.D. | Mitochondrial inner membrane; Ca^2+^ sensor that regulates MCU | ([Alevriadou et al., 2017](#_ENREF_2)) |
| MICU2 | *MICU2* | Controls MCU activity | 43.69 | N.D. | Mitochondrial inner membrane; Ca^2+^ sensor that regulates MCU | ([Alevriadou et al., 2017](#_ENREF_2)) |
| MICU3 | *MICU3* | Controls MCU activity | 3.16 | N.D. | Mitochondrial inner membrane; Ca^2+^ sensor that regulates MCU | ([Alevriadou et al., 2017](#_ENREF_2)) |
| MCUR1 | *MCUR1* | Controls MCU activity | 14.56 | N.D. | Mitochondrial inner membrane; Ca^2+^ sensor that regulates MCU | ([Alevriadou et al., 2017](#_ENREF_2)) |
| MITOK | *CCDC51* | K^+^ | 13.47 | NO production and NVC** | Mitochondrial inner membrane; A reduction in cytosolic ATP | ([Katakam et al., 2013](#_ENREF_62); [Paggio et al., 2019](#_ENREF_106)) |
| VDAC1 | *VDAC1* | Metabolites and ions (anions and cations) | 181.41 | Oxidative metabolism | Mitochondrial outer membrane | ([Alexander et al., 2023a](#_ENREF_3); [Rocca et al., 2023](#_ENREF_115)) |
| VDAC2 | *VDAC2* | Metabolites and ions (anions and cations) | 175.78 | Oxidative metabolism | Mitochondrial outer membrane | ([Alexander et al., 2023a](#_ENREF_3); [Rocca et al., 2023](#_ENREF_115)) |
| VDAC3 | *VDAC3* | Metabolites and ions (anions and cations) | 131.41 | Oxidative metabolism | Mitochondrial outer membrane | ([Alexander et al., 2023a](#_ENREF_3); [Rocca et al., 2023](#_ENREF_115)) |
| GHITM or TBIM5 | *GHITM* | Ca^2+^/H^+^ exchanger | 108.12 | N.D. | Mitochondrial inner membrane | ([Austin et al., 2022](#_ENREF_9)) |
| SMDT1 | *SMDT1* | Regulator of MCU | 50.42 | N.D. | Mitochondrial inner membrane | ([Sancak et al., 2013](#_ENREF_118)) |
| Cx43 | *GJA1* | Molecules of up to 1,000 Daltons | 85.27 | Expressed in hCMEC/D3 cells*  BBB signaling and homeostasis** | PM and Interendothelial junctions | ([Kaneko et al., 2015](#_ENREF_59); [Bader et al., 2017](#_ENREF_11); [Hoorelbeke et al., 2020](#_ENREF_53); [Phillips et al., 2023](#_ENREF_111)) |
| Cx45 | *GJC1* | Molecules of up to 1,000 Daltons | 16.81 | Expressed in hCMEC/D3 cells* | Interendothelial junctions | ([Bader et al., 2017](#_ENREF_11); [Davis et al., 2024](#_ENREF_34)) |
| Cx47 | *GJC2* | Molecules of up to 1,000 Daltons | 2.12 | N.D. | Interendothelial junctions | ([Ferrell et al., 2010](#_ENREF_41)) |
| Pannexin 1 | *PANX1* | Molecules of up to 1,500 Daltons (also regarded as ATP efflux systems) | 12.20 | Expressed in hCMEC/D3 cells* | PM; depolarization, extracellular K^+^, intracellular Ca^2+^ increase, lipids, mechanical stress | ([Kaneko et al., 2015](#_ENREF_59); [Navis et al., 2020](#_ENREF_99)) |
| Pannexin 2 | *PANX2* | Anions and cations, with a slight anion preference | 1.41 | N.D. | PM, ER, Golgi apparatus, MAMs; ER Ca^2+^ leakage? | ([Le Vasseur et al., 2019](#_ENREF_71); [He et al., 2023](#_ENREF_50)) |

Abbreviations: ASIC: acid-sensing ion channel; BK_Ca_: big-conductance Ca^2+^-activated K^+^ channel; CaCC: Ca^2+^-activated Cl^-^ channel; CAHLM5: Calcium Homeostasis Modulator Family Member 5; CaMKII: Ca^2+^/Calmodulin-dependent protein kinase II; CatSper1: cation channel, sperm associated 1; CCL1: CLIC-like chloride channel 1; ClC-2: Cl^-^ channel 2; CLIC1-4: Cl^-^ intracellular channel 1-4; EL: endolysosomal; ENaC: epithelial Na^+^ channel; ER: endoplasmic reticulum; FAIM2: Fas Apoptotic Inhibitory Molecule 2; GHITM: Single-Pass Membrane Protein With Aspartate Rich Tail 1; GlyR: glycine receptor; HCN2-3; hyperpolarization-activated cyclic nucleotide-gated 2-3; LRRC8A: leucine-rich repeat-containing 8A; MAMs: mitochondria-associated ER membranes; MERCS: mitochondria-endoplasmic reticulum contact sites; nAchR: nicotinic acetylcholine receptor; NALCN: sodium leakage channel; PACC1: proton-activated chloride channel; PIP(3,5)P_2_: phosphatidylinositol-3,5-bisphosphate; ROS: reactive oxygen species; SK_Ca_: small-conductance Ca^2+^-activated K^+^ channel; SFK: Src family kinase; SMDT: Single-Pass Membrane Protein With Aspartate Rich Tail 1; TMBIM1: transmembrane BAX inhibitor motif containing 1; TMBIM4: transmembrane BAX inhibitor motif containing 4; TMBIM5: transmembrane BAX inhibitor motif containing 5; TMBIM6: transmembrane BAX inhibitor motif containing 6; TMC-7: transmembrane channel-like 7; TMOC1: Transmembrane and coiled-coil domain 1; TMEM9: transmembrane protein 9; TMEM120A: transmembrane protein 120A; TREK-1: TWIK-related K^+^ channel 1; TRPC: transient receptor potential canonical; TRPM: transient receptor potential melastatin; TRPV: transient receptor potential vanilloid; TWIK-2: two-pore domain potassium channel-2; V_M_: resting membrane potential; VRAC: volume-regulated anion channel.

*Evidence from studies carried out in hCMEC/D3 cells.

**Putative role played by a given ion channel, based on findings reported in other cellular models of the BBB.

**Supplementary Table 4. Ion channel subunits expressed by hCMEC/D3 cells**

| **Subunit name** | **Gene** | **mRNA average counts** | **Function in hCMEC/D3cells** | **Functional role** | **Reference(s)** |
| --- | --- | --- | --- | --- | --- |
| K^+^ Voltage-Gated Channel Subfamily A Regulatory β Subunit 2 | *KCNAB2* | 12.75 | N.D. | Modulates the gating and kinetics of K_V_1/K_V_2 channels, membrane localization, and their redox sensitivity | ([Bahring et al., 2001](#_ENREF_12); [McCormack et al., 2002](#_ENREF_88); [Tipparaju et al., 2008](#_ENREF_130)) |
| K^+^ Voltage-Gated Channel Subfamily A Regulatory β Subunit 3 | *KCNAB3* | 2.62 | N.D. | Modulates the gating and kinetics of K_V_1/K_V_2 channels, membrane localization, and their redox sensitivity | ([Heinemann et al., 1995](#_ENREF_51); [Leicher et al., 1998](#_ENREF_72)) |
| KChIP2  (K^+^ Voltage-Gated Channel Interacting Protein 2) | *KCNIP2* | 1.01 | N.D. | Modulates K_V_4 channel expression at the cell membrane, gating characteristics, inactivation kinetics and rate of recovery from inactivation | ([Wu et al., 2023](#_ENREF_137)) |
| KChIP3  (K^+^ Voltage-Gated Channel Interacting Protein 3 - Calsenilin) | *KCNIP3* | 1.20 | N.D. | Modulates K_V_4 channel expression at the cell membrane, gating characteristics, inactivation kinetics and rate of recovery from inactivation | ([Wu et al., 2023](#_ENREF_137)) |
| Na^+^ Voltage-Gated Channel β Subunit 1 | *SCN1B* | 26.79 | N.D. | Modulates the gating properties, subcellular location and kinetics of Na_V_ channels | ([Al-Ward et al., 2020](#_ENREF_1)) |
| Ca^2+^ Voltage-Gated Channel Auxiliary Subunit β 1 | *CACNB1* | 7.84 | N.D. | Regulates the expression of Ca_V_ channels on the plasma membrane | ([Catterall, 2011](#_ENREF_28)) |
| Ca^2+^ Voltage-Gated Channel Auxiliary Subunit β 3 | *CACNB3* | 29.67 | BBB permeability* | Modulates ER Ca^2+^ release through InsP_3_Rs | ([Martus et al., 2024](#_ENREF_87)) |
| Ca^2+^ Voltage-Gated Channel Auxiliary α2δ1 | *CACNA2D1* | 6.37 | N.D. | Modulates Ca^2+^ current density and activation/inactivation kinetics of the Ca_V_ channel | ([Catterall, 2011](#_ENREF_28)) |
| Ca^2+^ Voltage-Gated Channel Auxiliary α2δ4 | *CACNA2D4* | 3.12 | N.D. | Modulates Ca^2+^ current density and activation/inactivation kinetics of the Ca_V_ channel | ([Catterall, 2011](#_ENREF_28)) |
| Ca^2+^ Voltage-Gated Channel Auxiliary Subunit γ 6 | *CACNG6* | 7.99 | N.D. | Modulates the voltage-dependence of Ca_V_1.2 channels | ([Catterall, 2011](#_ENREF_28)) |
| Ca^2+^ Voltage-Gated Channel Auxiliary Subunit γ 8 | *CACNG8* | 1.53 | N.D. | Modulates the voltage-dependence of Ca_V_1.2 channels | ([Catterall, 2011](#_ENREF_28)) |
| STIM1 | *STIM1* | 27.18 | N.D. | ER Ca^2+^ sensor | ([Moccia et al., 2023a](#_ENREF_93)) |
| STIM 2 | *STIM2* | 10.55 | Controls SOCE activation* | ER Ca^2+^ sensor | ([Zuccolo et al., 2019b](#_ENREF_152)) |

Abbreviations: BBB: blood-brain barrier; Ca_V_: voltage-gated Ca^2+^ channels; K_V_: voltage-gated K^+^ channels; Na_V_: voltage-gated Na^+^ channels; STIM1: stromal interaction molecule 1; STIM2; stromal interaction molecule 2.

*Evidence from studies carried out in hCMEC/D3 cells.

**Supplementary Table 5. GPCRs expressed in hCMEC/D3 cells**

| **GPCR name** | **Gene** | **Agonist** | **mRNA average counts** | **Functional role in hCMEC/D3 cells** | **Type of G protein** | **Reference (s)** |
| --- | --- | --- | --- | --- | --- | --- |
| Adenosine A1 receptor | *ADORA1* | Adenosine | 1.02 | Expressed in hCMEC/D3 cells, unknown function | G_i_ | ([Bader et al., 2017](#_ENREF_11); [Alexander et al., 2023a](#_ENREF_3)) |
| Adenosine A2a receptor | *ADORA2A* | Adenosine | 3.49 | BBB permeability | G_s_ | ([Bader et al., 2017](#_ENREF_11); [Alexander et al., 2023a](#_ENREF_3)) |
| Adenosine A2b receptor | *ADORA2B* | Adenosine | 18.03 | BBB permeability* | G_s_ | ([Mills et al., 2011](#_ENREF_91); [Bader et al., 2017](#_ENREF_11); [Alexander et al., 2023a](#_ENREF_3)) |
| GABA_B_ receptor subunit 1 | *GABBR1* | GABA | 14.46 | Ca^2+^ signaling* | G_i/o_ | ([Negri et al., 2022](#_ENREF_104); [Alexander et al., 2023a](#_ENREF_3)) |
| α_1B_-adrenoreceptor | *ADRA1B* | Adrenaline > Noradrenaline | 6.05 | Ca^2+^ signaling? | G_q_ | ([Alexander et al., 2023a](#_ENREF_3)) |
| β_1_-adrenoreceptor | *ADRB1* | Noradrenaline > Adrenaline | 2.37 | Increase in CBF** | G_s_ | ([Asano et al., 2020](#_ENREF_8); [Alexander et al., 2023a](#_ENREF_3)) |
| β_2_-adrenoreceptor | *ADRB2* | Adrenaline > Noradrenaline | 12.27 | BBB permeability** | G_s_ | ([Sun et al., 2017](#_ENREF_126); [Alexander et al., 2023a](#_ENREF_3)) |
| Histamine receptor H1 | *HRH1* | Histamine | 9.30 | Ca^2+^ signaling and NO release*, extracellular Na^+^ sensing | G_s_ | ([Berra-Romani et al., 2020](#_ENREF_16); [Brunetti et al., 2025](#_ENREF_25)) |
| Purinergic receptor P2Y2 | *P2RY2* | UTP>ATP | 1.97 | Ca^2+^ signaling* | G_q_ | ([Moccia et al., 2001](#_ENREF_92); [Bintig et al., 2012](#_ENREF_19)) |
| Purinergic receptor P2Y11 | *P2RY11* | ATP>ADP | 7.48 | Ca^2+^ signaling* | G_q_ | ([Bintig et al., 2012](#_ENREF_19)) |
| TMC6 | *TMC6* | Noxious heat | 12.40 | N.D. | G_q_ | ([Zhang et al., 2024a](#_ENREF_142)) |

Abbreviations: CBF: cerebral blood flow; GABA: γ-aminobutyric acid; BBB: blood-brain barrier; NO: nitric oxide; TMC6: transmembrane-like channel 6.

*Evidence from studies carried out in hCMEC/D3 cells.

**Putative role played by a given GPCR, based on findings reported in other cellular models of the BBB.

**Supplementary Table 6. RTKs expressed in hCMEC/D3 cells**

| **RTK name** | **Gene** | **Interacting Factor(s)** | **mRNA average counts** | **Functional role** | **Reference (s)** |
| --- | --- | --- | --- | --- | --- |
| FGFR-1 | *FGFR1* | FGF1-6,8,10,17,19-23 | 33.89 | BBB integrity**  BBB permeability** | ([Bader et al., 2017](#_ENREF_11); [Chen et al., 2020](#_ENREF_31); [Alexander et al., 2023a](#_ENREF_3); [Kriauciunaite et al., 2023](#_ENREF_67)) |
| FGFR-3 | *FGFR3* | FGF8-9 | 1.39 | BBB integrity** | ([Bader et al., 2017](#_ENREF_11); [Alexander et al., 2023a](#_ENREF_3); [Huang et al., 2024](#_ENREF_54)) |
| EGFR  Epidermal growth factor receptor | *EGFR* | EGF  TGF-α | 15.41 | BBB permeability* | ([Mills et al., 2011](#_ENREF_91); [Chen et al., 2015](#_ENREF_30); [Bader et al., 2017](#_ENREF_11); [Alexander et al., 2023a](#_ENREF_3)) |
| VEGFR-1 | *FLT1* | VEGF-B  PLCG1 | 3.81 | BBB permeability** | ([Schreurs et al., 2012](#_ENREF_122); [Salmeri et al., 2013](#_ENREF_117)) |
| VEGFR-2 | *KDR* | VEGFA; VEGF-C and VEGF-D after proteolytic cleavage | 4.16 | BBB permeability* and angiogenesis* | ([Yang et al., 2013](#_ENREF_140); [Apte et al., 2019](#_ENREF_7); [Zhang et al., 2019](#_ENREF_147); [Sandoval et al., 2025](#_ENREF_119)) |
| IGF1-R | *IGF1R* | IGF-1  IGF-2 | 16.04 | BBB integrity** | ([Higashi et al., 2020](#_ENREF_52); [Gulej et al., 2024](#_ENREF_45)) |
| PDGFR-B | *PDGFRB* | PDGF-B  PDGF-D | 9.36 | BBB integrity**  Pericytes attraction** | ([Shen et al., 2019](#_ENREF_123)) |

Abbreviations: EGFR: epidermal growth factor receptor; FGFR-1: Fibroblast growth factor receptor 1; FGFR-3: Fibroblast growth factor receptor 3; IGF1-R: Insulin-like growth factor 1 receptor; PDGFR-B; Platelet-derived growth factor receptor beta; VEGFR-1: vascular endothelial growth factor receptor-1.

*Evidence from studies carried out in hCMEC/D3 cells.

**Putative role played by a given RTK, based on findings reported in other cellular models of the BBB.

**References**

Al-Ward, H., Liu, C.Y., Liu, N., Shaher, F., Al-Nusaif, M., Mao, J., et al. (2020). Voltage-Gated Sodium Channel beta1 Gene: An Overview. *Hum Hered* 85(3-6)**,** 101-109. doi: 10.1159/000516388.

Alevriadou, B.R., Shanmughapriya, S., Patel, A., Stathopulos, P.B., and Madesh, M. (2017). Mitochondrial Ca(2+) transport in the endothelium: regulation by ions, redox signalling and mechanical forces. *J R Soc Interface* 14(137). doi: 10.1098/rsif.2017.0672.

Alexander, S.P.H., Christopoulos, A., Davenport, A.P., Kelly, E., Mathie, A.A., Peters, J.A., et al. (2023a). The Concise Guide to PHARMACOLOGY 2023/24: G protein-coupled receptors. *Br J Pharmacol* 180 Suppl 2**,** S23-S144. doi: 10.1111/bph.16177.

Alexander, S.P.H., Fabbro, D., Kelly, E., Mathie, A.A., Peters, J.A., Veale, E.L., et al. (2023b). The Concise Guide to PHARMACOLOGY 2023/24: Transporters. *Br J Pharmacol* 180 Suppl 2**,** S374-S469. doi: 10.1111/bph.16182.

Alexander, S.P.H., Mathie, A.A., Peters, J.A., Veale, E.L., Striessnig, J., Kelly, E., et al. (2023c). The Concise Guide to PHARMACOLOGY 2023/24: Ion channels. *Br J Pharmacol* 180 Suppl 2(Suppl 2)**,** S145-S222. doi: 10.1111/bph.16178.

Alquisiras-Burgos, I., Hernandez-Cruz, A., Peralta-Arrieta, I., and Aguilera, P. (2024). Resveratrol Prevents Cell Swelling Through Inhibition of SUR1 Expression in Brain Micro Endothelial Cells Subjected to OGD/Recovery. *Mol Neurobiol* 61(4)**,** 2099-2119. doi: 10.1007/s12035-023-03686-0.

Apte, R.S., Chen, D.S., and Ferrara, N. (2019). VEGF in Signaling and Disease: Beyond Discovery and Development. *Cell* 176(6)**,** 1248-1264. doi: 10.1016/j.cell.2019.01.021.

Asano, N., Hishiyama, S., Ishiyama, T., Kotoda, M., and Matsukawa, T. (2020). Effects of beta(1)-adrenergic receptor blockade on the cerebral microcirculation in the normal state and during global brain ischemia/reperfusion injury in rabbits. *BMC Pharmacol Toxicol* 21(1)**,** 13. doi: 10.1186/s40360-020-0394-7.

Austin, S., Mekis, R., Mohammed, S.E.M., Scalise, M., Wang, W.A., Galluccio, M., et al. (2022). TMBIM5 is the Ca(2+) /H(+) antiporter of mammalian mitochondria. *EMBO Rep* 23(12)**,** e54978. doi: 10.15252/embr.202254978.

Avalos Prado, P., Chassot, A.A., Landra-Willm, A., and Sandoz, G. (2022). Regulation of two-pore-domain potassium TREK channels and their involvement in pain perception and migraine. *Neurosci Lett* 773**,** 136494. doi: 10.1016/j.neulet.2022.136494.

Bader, A., Bintig, W., Begandt, D., Klett, A., Siller, I.G., Gregor, C., et al. (2017). Adenosine receptors regulate gap junction coupling of the human cerebral microvascular endothelial cells hCMEC/D3 by Ca(2+) influx through cyclic nucleotide-gated channels. *J Physiol* 595(8)**,** 2497-2517. doi: 10.1113/JP273150.

Bahring, R., Milligan, C.J., Vardanyan, V., Engeland, B., Young, B.A., Dannenberg, J., et al. (2001). Coupling of voltage-dependent potassium channel inactivation and oxidoreductase active site of Kvbeta subunits. *J Biol Chem* 276(25)**,** 22923-22929. doi: 10.1074/jbc.M100483200.

Balbuena, P., Li, W., Rzigalinski, B.A., and Ehrich, M. (2012). Malathion/oxon and lead acetate increase gene expression and protein levels of transient receptor potential canonical channel subunits TRPC1 and TRPC4 in rat endothelial cells of the blood-brain barrier. *Int J Toxicol* 31(3)**,** 238-249. doi: 10.1177/1091581812442688.

Berra-Romani, R., Brunetti, V., Pellavio, G., Soda, T., Laforenza, U., Scarpellino, G., et al. (2023). Allyl Isothiocianate Induces Ca(2+) Signals and Nitric Oxide Release by Inducing Reactive Oxygen Species Production in the Human Cerebrovascular Endothelial Cell Line hCMEC/D3. *Cells* 12(13)**,** 1732. doi: 10.3390/cells12131732.

Berra-Romani, R., Faris, P., Negri, S., Botta, L., Genova, T., and Moccia, F. (2019). Arachidonic Acid Evokes an Increase in Intracellular Ca(2+) Concentration and Nitric Oxide Production in Endothelial Cells from Human Brain Microcirculation. *Cells* 8(7). doi: 10.3390/cells8070689.

Berra-Romani, R., Faris, P., Pellavio, G., Orgiu, M., Negri, S., Forcaia, G., et al. (2020). Histamine induces intracellular Ca(2+) oscillations and nitric oxide release in endothelial cells from brain microvascular circulation. *J Cell Physiol* 235(2)**,** 1515-1530. doi: 10.1002/jcp.29071.

Berrout, J., Jin, M., and O'Neil, R.G. (2012). Critical role of TRPP2 and TRPC1 channels in stretch-induced injury of blood-brain barrier endothelial cells. *Brain Res* 1436**,** 1-12. doi: 10.1016/j.brainres.2011.11.044.

Bertelli, S., Remigante, A., Zuccolini, P., Barbieri, R., Ferrera, L., Picco, C., et al. (2021). Mechanisms of Activation of LRRC8 Volume Regulated Anion Channels. *Cell Physiol Biochem* 55(S1)**,** 41-56. doi: 10.33594/000000329.

Bintig, W., Begandt, D., Schlingmann, B., Gerhard, L., Pangalos, M., Dreyer, L., et al. (2012). Purine receptors and Ca(2+) signalling in the human blood-brain barrier endothelial cell line hCMEC/D3. *Purinergic Signal* 8(1)**,** 71-80. doi: 10.1007/s11302-011-9262-7.

Bittner, S., Ruck, T., Schuhmann, M.K., Herrmann, A.M., Moha ou Maati, H., Bobak, N., et al. (2013). Endothelial TWIK-related potassium channel-1 (TREK1) regulates immune-cell trafficking into the CNS. *Nat Med* 19(9)**,** 1161-1165. doi: 10.1038/nm.3303.

Bobak, N., Feliciangeli, S., Chen, C.C., Ben Soussia, I., Bittner, S., Pagnotta, S., et al. (2017). Recombinant tandem of pore-domains in a Weakly Inward rectifying K(+) channel 2 (TWIK2) forms active lysosomal channels. *Sci Rep* 7(1)**,** 649. doi: 10.1038/s41598-017-00640-8.

Boletta, A., and Caplan, M.J. (2025). Physiologic mechanisms underlying polycystic kidney disease. *Physiol Rev* 105(3)**,** 1553-1607. doi: 10.1152/physrev.00018.2024.

Bonson, G., Lambert, A.R., Sackheim, A.M., Howard, A.J., Piffard, S.H., Lescieur-Garcia, C., et al. (2025). Endothelial-Specific Knockout of the Scramblase Tmem16f Impairs in Vivo Clot Formation. *Shock* 63(5)**,** 788-795. doi: 10.1097/SHK.0000000000002553.

Brunetti, V., Berra-Romani, R., Conca, F., Soda, T., Biella, G.R., Gerbino, A., et al. (2024). Lysosomal TRPML1 triggers global Ca(2+) signals and nitric oxide release in human cerebrovascular endothelial cells. *Front Physiol* 15**,** 1426783. doi: 10.3389/fphys.2024.1426783.

Brunetti, V., Berra-Romani, R., Coyotl-Santiago, N., Esquitin-Gonzalez, Y., Chinigo, G., Biella, G.R., et al. (2025). Histamine 1 receptors and reverse-mode Na(+)/Ca(2+) exchanger drive extracellular Na(+)-dependent intracellular Ca(2+) oscillations in human cerebrovascular endothelial cells. *Cell Calcium* 131**,** 103067. doi: 10.1016/j.ceca.2025.103067.

Bultynck, G., Kiviluoto, S., Henke, N., Ivanova, H., Schneider, L., Rybalchenko, V., et al. (2012). The C terminus of Bax inhibitor-1 forms a Ca2+-permeable channel pore. *J Biol Chem* 287(4)**,** 2544-2557. doi: 10.1074/jbc.M111.275354.

Bushell, S.R., Pike, A.C.W., Falzone, M.E., Rorsman, N.J.G., Ta, C.M., Corey, R.A., et al. (2019). The structural basis of lipid scrambling and inactivation in the endoplasmic reticulum scramblase TMEM16K. *Nat Commun* 10(1)**,** 3956. doi: 10.1038/s41467-019-11753-1.

Catterall, W.A. (2011). Voltage-gated calcium channels. *Cold Spring Harb Perspect Biol* 3(8)**,** a003947. doi: 10.1101/cshperspect.a003947.

Chen, G.L., Li, J.Y., Chen, X., Liu, J.W., Zhang, Q., Liu, J.Y., et al. (2024). Mechanosensitive channels TMEM63A and TMEM63B mediate lung inflation-induced surfactant secretion. *J Clin Invest* 134(5). doi: 10.1172/JCI174508.

Chen, L., Liu, W., Wang, P., Xue, Y., Su, Q., Zeng, C., et al. (2015). Endophilin-1 regulates blood-brain barrier permeability via EGFR-JNK signaling pathway. *Brain Res* 1606**,** 44-53. doi: 10.1016/j.brainres.2015.02.032.

Chen, P., Tang, H., Zhang, Q., Xu, L., Zhou, W., Hu, X., et al. (2020). Basic Fibroblast Growth Factor (bFGF) Protects the Blood-Brain Barrier by Binding of FGFR1 and Activating the ERK Signaling Pathway After Intra-Abdominal Hypertension and Traumatic Brain Injury. *Med Sci Monit* 26**,** e922009. doi: 10.12659/MSM.922009.

Chen, W., Paradkar, P.N., Li, L., Pierce, E.L., Langer, N.B., Takahashi-Makise, N., et al. (2009). Abcb10 physically interacts with mitoferrin-1 (Slc25a37) to enhance its stability and function in the erythroid mitochondria. *Proc Natl Acad Sci U S A* 106(38)**,** 16263-16268. doi: 10.1073/pnas.0904519106.

Dang, X., Eliceiri, B.P., Baird, A., and Costantini, T.W. (2015). CHRFAM7A: a human-specific alpha7-nicotinic acetylcholine receptor gene shows differential responsiveness of human intestinal epithelial cells to LPS. *FASEB J* 29(6)**,** 2292-2302. doi: 10.1096/fj.14-268037.

Davis, M.J., Castorena-Gonzalez, J.A., Li, M., Zawieja, S.D., Simon, A.M., Geng, X., et al. (2024). Connexin-45 is expressed in mouse lymphatic endothelium and required for lymphatic valve function. *JCI Insight* 9(16). doi: 10.1172/jci.insight.169931.

DeCoursey, T.E. (2018). Voltage and pH sensing by the voltage-gated proton channel, H(V)1. *J R Soc Interface* 15(141). doi: 10.1098/rsif.2018.0108.

Delaunay, A., Gasull, X., Salinas, M., Noel, J., Friend, V., Lingueglia, E., et al. (2012). Human ASIC3 channel dynamically adapts its activity to sense the extracellular pH in both acidic and alkaline directions. *Proc Natl Acad Sci U S A* 109(32)**,** 13124-13129. doi: 10.1073/pnas.1120350109.

Ding, R., Yin, Y.L., and Jiang, L.H. (2021). Reactive Oxygen Species-Induced TRPM2-Mediated Ca(2+) Signalling in Endothelial Cells. *Antioxidants (Basel)* 10(5). doi: 10.3390/antiox10050718.

Dragoni, S., Moccia, F., and Bootman, M.D. (2025). The Roles of Transient Receptor Potential (TRP) Channels Underlying Aberrant Calcium Signaling in Blood-Retinal Barrier Dysfunction. *Cold Spring Harb Perspect Biol* 17(2). doi: 10.1101/cshperspect.a041763.

Duran, C., Qu, Z., Osunkoya, A.O., Cui, Y., and Hartzell, H.C. (2012). ANOs 3-7 in the anoctamin/Tmem16 Cl- channel family are intracellular proteins. *Am J Physiol Cell Physiol* 302(3)**,** C482-493. doi: 10.1152/ajpcell.00140.2011.

Earley, S. (2011). Endothelium-dependent cerebral artery dilation mediated by transient receptor potential and Ca2+-activated K+ channels. *J Cardiovasc Pharmacol* 57(2)**,** 148-153. doi: 10.1097/FJC.0b013e3181f580d9.

Ferrell, R.E., Baty, C.J., Kimak, M.A., Karlsson, J.M., Lawrence, E.C., Franke-Snyder, M., et al. (2010). GJC2 missense mutations cause human lymphedema. *Am J Hum Genet* 86(6)**,** 943-948. doi: 10.1016/j.ajhg.2010.04.010.

Finbow, M.E., and Harrison, M.A. (1997). The vacuolar H+-ATPase: a universal proton pump of eukaryotes. *Biochem J* 324 ( Pt 3)(Pt 3)**,** 697-712. doi: 10.1042/bj3240697.

Furst, J., Botta, G., Saino, S., Dopinto, S., Gandini, R., Dossena, S., et al. (2006). The ICln interactome. *Acta Physiol (Oxf)* 187(1-2)**,** 43-49. doi: 10.1111/j.1748-1716.2006.01549.x.

Ganapathi, M., Friocourt, G., Gueguen, N., Friederich, M.W., Le Gac, G., Okur, V., et al. (2022). A homozygous splice variant in ATP5PO, disrupts mitochondrial complex V function and causes Leigh syndrome in two unrelated families. *J Inherit Metab Dis* 45(5)**,** 996-1012. doi: 10.1002/jimd.12526.

Gulej, R., Csik, B., Faakye, J., Tarantini, S., Shanmugarama, S., Chandragiri, S.S., et al. (2024). Endothelial deficiency of insulin-like growth factor-1 receptor leads to blood-brain barrier disruption and accelerated endothelial senescence in mice, mimicking aspects of the brain aging phenotype. *Microcirculation* 31(2)**,** e12840. doi: 10.1111/micc.12840.

Guo, L., Mao, Q., He, J., Liu, X., Piao, X., Luo, L., et al. (2023). Disruption of ER ion homeostasis maintained by an ER anion channel CLCC1 contributes to ALS-like pathologies. *Cell Res* 33(7)**,** 497-515. doi: 10.1038/s41422-023-00798-z.

Gururaja Rao, S., Ponnalagu, D., Patel, N.J., and Singh, H. (2018). Three Decades of Chloride Intracellular Channel Proteins: From Organelle to Organ Physiology. *Curr Protoc Pharmacol* 80(1)**,** 11 21 11-11 21 17. doi: 10.1002/cpph.36.

Harraz, O.F., Klug, N.R., Senatore, A.J., Hill-Eubanks, D.C., and Nelson, M.T. (2022). Piezo1 Is a Mechanosensor Channel in Central Nervous System Capillaries. *Circ Res* 130(10)**,** 1531-1546. doi: 10.1161/CIRCRESAHA.122.320827.

Harraz, O.F., Longden, T.A., Hill-Eubanks, D., and Nelson, M.T. (2018). PIP2 depletion promotes TRPV4 channel activity in mouse brain capillary endothelial cells. *Elife* 7**,** e38689. doi: 10.7554/eLife.38689.

He, Z., Zhao, Y., Rau, M.J., Fitzpatrick, J.A.J., Sah, R., Hu, H., et al. (2023). Structural and functional analysis of human pannexin 2 channel. *Nat Commun* 14(1)**,** 1712. doi: 10.1038/s41467-023-37413-z.

Heinemann, S.H., Rettig, J., Wunder, F., and Pongs, O. (1995). Molecular and functional characterization of a rat brain Kv beta 3 potassium channel subunit. *FEBS Lett* 377(3)**,** 383-389. doi: 10.1016/0014-5793(95)01377-6.

Higashi, Y., Sukhanov, S., Shai, S.Y., Danchuk, S., Snarski, P., Li, Z., et al. (2020). Endothelial deficiency of insulin-like growth factor-1 receptor reduces endothelial barrier function and promotes atherosclerosis in Apoe-deficient mice. *Am J Physiol Heart Circ Physiol* 319(4)**,** H730-H743. doi: 10.1152/ajpheart.00064.2020.

Hoorelbeke, D., Decrock, E., De Smet, M., De Bock, M., Descamps, B., Van Haver, V., et al. (2020). Cx43 channels and signaling via IP(3)/Ca(2+), ATP, and ROS/NO propagate radiation-induced DNA damage to non-irradiated brain microvascular endothelial cells. *Cell Death Dis* 11(3)**,** 194. doi: 10.1038/s41419-020-2392-5.

Huang, W.T., Chen, X.J., Lin, Y.K., Shi, J.F., Li, H., Wu, H.D., et al. (2024). FGF17 protects cerebral ischemia reperfusion-induced blood-brain barrier disruption via FGF receptor 3-mediated PI3K/AKT signaling pathway. *Eur J Pharmacol* 971**,** 176521. doi: 10.1016/j.ejphar.2024.176521.

Jentsch, T.J., and Pusch, M. (2018). CLC Chloride Channels and Transporters: Structure, Function, Physiology, and Disease. *Physiol Rev* 98(3)**,** 1493-1590. doi: 10.1152/physrev.00047.2017.

Jeschke, J.K., Biagioni, C., Schierling, T., Wagner, I.V., Borgel, F., Schepmann, D., et al. (2021). The Action of Reproductive Fluids and Contained Steroids, Prostaglandins, and Zn(2+) on CatSper Ca(2+) Channels in Human Sperm. *Front Cell Dev Biol* 9**,** 699554. doi: 10.3389/fcell.2021.699554.

Kageyama, T., Shimizu, T., Shirai, K., Nabeshima, S., Ozawa, S., Fujii, T., et al. (2025). Ion Channel Function of Human TMEM16F Is Associated with Phospholipid Transport through Its Subunit Cavity. *Biol Pharm Bull* 48(5)**,** 595-605. doi: 10.1248/bpb.b24-00859.

Kambe, T., Tsuji, T., Hashimoto, A., and Itsumura, N. (2015). The Physiological, Biochemical, and Molecular Roles of Zinc Transporters in Zinc Homeostasis and Metabolism. *Physiol Rev* 95(3)**,** 749-784. doi: 10.1152/physrev.00035.2014.

Kaneko, Y., Tachikawa, M., Akaogi, R., Fujimoto, K., Ishibashi, M., Uchida, Y., et al. (2015). Contribution of pannexin 1 and connexin 43 hemichannels to extracellular calcium-dependent transport dynamics in human blood-brain barrier endothelial cells. *J Pharmacol Exp Ther* 353(1)**,** 192-200. doi: 10.1124/jpet.114.220210.

Kang, H., and Lee, C.J. (2024). Transmembrane proteins with unknown function (TMEMs) as ion channels: electrophysiological properties, structure, and pathophysiological roles. *Exp Mol Med* 56(4)**,** 850-860. doi: 10.1038/s12276-024-01206-1.

Karakas, E., Strange, K., and Denton, J.S. (2025). Recent advances in structural characterization of volume-regulated anion channels (VRACs). *J Physiol* 603(15)**,** 4201-4211. doi: 10.1113/JP286189.

Katakam, P.V., Wappler, E.A., Katz, P.S., Rutkai, I., Institoris, A., Domoki, F., et al. (2013). Depolarization of mitochondria in endothelial cells promotes cerebral artery vasodilation by activation of nitric oxide synthase. *Arterioscler Thromb Vasc Biol* 33(4)**,** 752-759. doi: 10.1161/ATVBAHA.112.300560.

Kern, D.M., Bleier, J., Mukherjee, S., Hill, J.M., Kossiakoff, A.A., Isacoff, E.Y., et al. (2023). Structural basis for assembly and lipid-mediated gating of LRRC8A:C volume-regulated anion channels. *Nat Struct Mol Biol* 30(6)**,** 841-852. doi: 10.1038/s41594-023-00944-6.

Kim, H.R., Lee, G.H., Ha, K.C., Ahn, T., Moon, J.Y., Lee, B.J., et al. (2008). Bax Inhibitor-1 Is a pH-dependent regulator of Ca2+ channel activity in the endoplasmic reticulum. *J Biol Chem* 283(23)**,** 15946-15955. doi: 10.1074/jbc.M800075200.

Kirichok, Y., Navarro, B., and Clapham, D.E. (2006). Whole-cell patch-clamp measurements of spermatozoa reveal an alkaline-activated Ca2+ channel. *Nature* 439(7077)**,** 737-740. doi: 10.1038/nature04417.

Kolisek, M., Nestler, A., Vormann, J., and Schweigel-Rontgen, M. (2012). Human gene SLC41A1 encodes for the Na+/Mg(2)+ exchanger. *Am J Physiol Cell Physiol* 302(1)**,** C318-326. doi: 10.1152/ajpcell.00289.2011.

Kriauciunaite, K., Pociute, A., Kausyle, A., Verkhratsky, A., and Pivoriunas, A. (2023). Basic Fibroblast Growth Factor Opens and Closes the Endothelial Blood-Brain Barrier in a Concentration-Dependent Manner. *Neurochem Res* 48(4)**,** 1211-1221. doi: 10.1007/s11064-022-03678-x.

Kunzelmann, K., Ousingsawat, J., and Schreiber, R. (2024). VSI: The anoctamins: Structure and function: "Intracellular" anoctamins. *Cell Calcium* 120**,** 102888. doi: 10.1016/j.ceca.2024.102888.

Lai, Y., Zhang, Y., Zhou, S., Xu, J., Du, Z., Feng, Z., et al. (2023). Structure of the human ATP synthase. *Mol Cell* 83(12)**,** 2137-2147 e2134. doi: 10.1016/j.molcel.2023.04.029.

Le, S.C., and Yang, H. (2021). Structure-Function of TMEM16 Ion Channels and Lipid Scramblases. *Adv Exp Med Biol* 1349**,** 87-109. doi: 10.1007/978-981-16-4254-8_6.

Le Vasseur, M., Chen, V.C., Huang, K., Vogl, W.A., and Naus, C.C. (2019). Pannexin 2 Localizes at ER-Mitochondria Contact Sites. *Cancers (Basel)* 11(3). doi: 10.3390/cancers11030343.

Leicher, T., Bahring, R., Isbrandt, D., and Pongs, O. (1998). Coexpression of the KCNA3B gene product with Kv1.5 leads to a novel A-type potassium channel. *J Biol Chem* 273(52)**,** 35095-35101. doi: 10.1074/jbc.273.52.35095.

Lim, X.R., Abd-Alhaseeb, M.M., Ippolito, M., Koide, M., Senatore, A.J., Plante, C., et al. (2024). Endothelial Piezo1 channel mediates mechano-feedback control of brain blood flow. *Nat Commun* 15(1)**,** 8686. doi: 10.1038/s41467-024-52969-0.

Lin, L.H., Jin, J., Nashelsky, M.B., and Talman, W.T. (2014). Acid-sensing ion channel 1 and nitric oxide synthase are in adjacent layers in the wall of rat and human cerebral arteries. *J Chem Neuroanat* 61-62**,** 161-168. doi: 10.1016/j.jchemneu.2014.10.002.

Lisak, D.A., Schacht, T., Enders, V., Habicht, J., Kiviluoto, S., Schneider, J., et al. (2015). The transmembrane Bax inhibitor motif (TMBIM) containing protein family: Tissue expression, intracellular localization and effects on the ER CA(2)(+)-filling state. *Biochim Biophys Acta* 1853(9)**,** 2104-2114. doi: 10.1016/j.bbamcr.2015.03.002.

Littler, D.R., Assaad, N.N., Harrop, S.J., Brown, L.J., Pankhurst, G.J., Luciani, P., et al. (2005). Crystal structure of the soluble form of the redox-regulated chloride ion channel protein CLIC4. *FEBS J* 272(19)**,** 4996-5007. doi: 10.1111/j.1742-4658.2005.04909.x.

Liu, J., Wan, F., Jin, Q., Li, X., Bhat, E.A., Guo, J., et al. (2020). Cryo-EM structures of human calcium homeostasis modulator 5. *Cell Discov* 6(1)**,** 81. doi: 10.1038/s41421-020-00228-z.

Liu, W., Li, Y., Bao, Y., and Tan, Z.Y. (2025). Lysosomal ion channels and pain. *Pain Rep* 10(4)**,** e1282. doi: 10.1097/PR9.0000000000001282.

Lloyd, E.E., Marrelli, S.P., Namiranian, K., and Bryan, R.M., Jr. (2009). Characterization of TWIK-2, a two-pore domain K+ channel, cloned from the rat middle cerebral artery. *Exp Biol Med (Maywood)* 234(12)**,** 1493-1502. doi: 10.3181/0903-RM-110.

Longden, T.A., Dabertrand, F., Koide, M., Gonzales, A.L., Tykocki, N.R., Brayden, J.E., et al. (2017). Capillary K(+)-sensing initiates retrograde hyperpolarization to increase local cerebral blood flow. *Nat Neurosci* 20(5)**,** 717-726. doi: 10.1038/nn.4533.

Lucitti, J.L., Tarte, N.J., and Faber, J.E. (2015). Chloride intracellular channel 4 is required for maturation of the cerebral collateral circulation. *Am J Physiol Heart Circ Physiol* 309(7)**,** H1141-1150. doi: 10.1152/ajpheart.00451.2015.

Luo, H., Rossi, E., Saubamea, B., Chasseigneaux, S., Cochois, V., Choublier, N., et al. (2019). Cannabidiol Increases Proliferation, Migration, Tubulogenesis, and Integrity of Human Brain Endothelial Cells through TRPV2 Activation. *Mol Pharm* 16(3)**,** 1312-1326. doi: 10.1021/acs.molpharmaceut.8b01252.

Luo, H., Saubamea, B., Chasseigneaux, S., Cochois, V., Smirnova, M., Glacial, F., et al. (2020). Molecular and Functional Study of Transient Receptor Potential Vanilloid 1-4 at the Rat and Human Blood-Brain Barrier Reveals Interspecies Differences. *Front Cell Dev Biol* 8**,** 578514. doi: 10.3389/fcell.2020.578514.

Lutsenko, S., LeShane, E.S., and Shinde, U. (2007). Biochemical basis of regulation of human copper-transporting ATPases. *Arch Biochem Biophys* 463(2)**,** 134-148. doi: 10.1016/j.abb.2007.04.013.

Ma, P., Huang, N., Tang, J., Zhou, Z., Xu, J., Chen, Y., et al. (2023). The TRPM4 channel inhibitor 9-phenanthrol alleviates cerebral edema after traumatic brain injury in rats. *Front Pharmacol* 14**,** 1098228. doi: 10.3389/fphar.2023.1098228.

Ma, Z., Tanis, J.E., Taruno, A., and Foskett, J.K. (2016). Calcium homeostasis modulator (CALHM) ion channels. *Pflugers Arch* 468(3)**,** 395-403. doi: 10.1007/s00424-015-1757-6.

Martus, D., Williams, S.K., Pichi, K., Mannebach-Gotz, S., Kaiser, N., Wardas, B., et al. (2024). Cavbeta3 Contributes to the Maintenance of the Blood-Brain Barrier and Alleviates Symptoms of Experimental Autoimmune Encephalomyelitis. *Arterioscler Thromb Vasc Biol* 44(8)**,** 1833-1851. doi: 10.1161/ATVBAHA.124.321141.

McCormack, K., Connor, J.X., Zhou, L., Ho, L.L., Ganetzky, B., Chiu, S.Y., et al. (2002). Genetic analysis of the mammalian K+ channel beta subunit Kvbeta 2 (Kcnab2). *J Biol Chem* 277(15)**,** 13219-13228. doi: 10.1074/jbc.M111465200.

McKenna, M.J., Sim, S.I., Ordureau, A., Wei, L., Harper, J.W., Shao, S., et al. (2020). The endoplasmic reticulum P5A-ATPase is a transmembrane helix dislocase. *Science* 369(6511). doi: 10.1126/science.abc5809.

Miller, T.J., and Davis, P.B. (2008). FXYD5 modulates Na+ absorption and is increased in cystic fibrosis airway epithelia. *Am J Physiol Lung Cell Mol Physiol* 294(4)**,** L654-664. doi: 10.1152/ajplung.00430.2007.

Mills, J.H., Alabanza, L., Weksler, B.B., Couraud, P.O., Romero, I.A., and Bynoe, M.S. (2011). Human brain endothelial cells are responsive to adenosine receptor activation. *Purinergic Signal* 7(2)**,** 265-273. doi: 10.1007/s11302-011-9222-2.

Moccia, F., Baruffi, S., Spaggiari, S., Coltrini, D., Berra-Romani, R., Signorelli, S., et al. (2001). P2y1 and P2y2 receptor-operated Ca2+ signals in primary cultures of cardiac microvascular endothelial cells. *Microvasc Res* 61(3)**,** 240-252. doi: 10.1006/mvre.2001.2306.

Moccia, F., Brunetti, V., Perna, A., Guerra, G., Soda, T., and Berra-Romani, R. (2023a). The Molecular Heterogeneity of Store-Operated Ca(2+) Entry in Vascular Endothelial Cells: The Different roles of Orai1 and TRPC1/TRPC4 Channels in the Transition from Ca(2+)-Selective to Non-Selective Cation Currents. *Int J Mol Sci* 24(4)**,** 3259. doi: 10.3390/ijms24043259.

Moccia, F., Brunetti, V., Soda, T., Berra-Romani, R., and Scarpellino, G. (2023b). Cracking the Endothelial Calcium (Ca(2+)) Code: A Matter of Timing and Spacing. *Int J Mol Sci* 24(23). doi: 10.3390/ijms242316765.

Moccia, F., and Dragoni, S. (2025). The Calcium Signalling Profile of the Inner Blood-Retinal Barrier in Diabetic Retinopathy. *Cells* 14(12). doi: 10.3390/cells14120856.

Moccia, F., Frost, C., Berra-Romani, R., Tanzi, F., and Adams, D.J. (2004). Expression and function of neuronal nicotinic ACh receptors in rat microvascular endothelial cells. *Am J Physiol Heart Circ Physiol* 286(2)**,** H486-491. doi: 10.1152/ajpheart.00620.2003.

Monteil, A., Guerineau, N.C., Gil-Nagel, A., Parra-Diaz, P., Lory, P., and Senatore, A. (2024). New insights into the physiology and pathophysiology of the atypical sodium leak channel NALCN. *Physiol Rev* 104(1)**,** 399-472. doi: 10.1152/physrev.00014.2022.

Mori, M.P., Lozoya, O.A., Brooks, A.M., Bortner, C.D., Nadalutti, C.A., Ryback, B., et al. (2025). Mitochondrial membrane hyperpolarization modulates nuclear DNA methylation and gene expression through phospholipid remodeling. *Nat Commun* 16(1)**,** 4029. doi: 10.1038/s41467-025-59427-5.

Navis, K.E., Fan, C.Y., Trang, T., Thompson, R.J., and Derksen, D.J. (2020). Pannexin 1 Channels as a Therapeutic Target: Structure, Inhibition, and Outlook. *ACS Chem Neurosci* 11(15)**,** 2163-2172. doi: 10.1021/acschemneuro.0c00333.

Neagoe, I., Stauber, T., Fidzinski, P., Bergsdorf, E.Y., and Jentsch, T.J. (2010). The late endosomal ClC-6 mediates proton/chloride countertransport in heterologous plasma membrane expression. *J Biol Chem* 285(28)**,** 21689-21697. doi: 10.1074/jbc.M110.125971.

Negri, S., Faris, P., Berra-Romani, R., Guerra, G., and Moccia, F. (2019). Endothelial Transient Receptor Potential Channels and Vascular Remodeling: Extracellular Ca(2 +) Entry for Angiogenesis, Arteriogenesis and Vasculogenesis. *Front Physiol* 10**,** 1618. doi: 10.3389/fphys.2019.01618.

Negri, S., Faris, P., Pellavio, G., Botta, L., Orgiu, M., Forcaia, G., et al. (2020a). Group 1 metabotropic glutamate receptors trigger glutamate-induced intracellular Ca(2+) signals and nitric oxide release in human brain microvascular endothelial cells. *Cell Mol Life Sci* 77(11)**,** 2235-2253. doi: 10.1007/s00018-019-03284-1.

Negri, S., Faris, P., Rosti, V., Antognazza, M.R., Lodola, F., and Moccia, F. (2020b). Endothelial TRPV1 as an Emerging Molecular Target to Promote Therapeutic Angiogenesis. *Cells* 9(6)**,** 1341. doi: 10.3390/cells9061341.

Negri, S., Scolari, F., Vismara, M., Brunetti, V., Faris, P., Terribile, G., et al. (2022). GABA(A) and GABA(B) Receptors Mediate GABA-Induced Intracellular Ca(2+) Signals in Human Brain Microvascular Endothelial Cells. *Cells* 11(23)**,** 3860. doi: 10.3390/cells11233860.

Owji, A.P., Kittredge, A., Zhang, Y., and Yang, T. (2021). Structure and Function of the Bestrophin family of calcium-activated chloride channels. *Channels (Austin)* 15(1)**,** 604-623. doi: 10.1080/19336950.2021.1981625.

Paggio, A., Checchetto, V., Campo, A., Menabo, R., Di Marco, G., Di Lisa, F., et al. (2019). Identification of an ATP-sensitive potassium channel in mitochondria. *Nature* 572(7771)**,** 609-613. doi: 10.1038/s41586-019-1498-3.

Paradkar, P.N., Zumbrennen, K.B., Paw, B.H., Ward, D.M., and Kaplan, J. (2009). Regulation of mitochondrial iron import through differential turnover of mitoferrin 1 and mitoferrin 2. *Mol Cell Biol* 29(4)**,** 1007-1016. doi: 10.1128/MCB.01685-08.

Park, L., Wang, G., Moore, J., Girouard, H., Zhou, P., Anrather, J., et al. (2014). The key role of transient receptor potential melastatin-2 channels in amyloid-beta-induced neurovascular dysfunction. *Nat Commun* 5**,** 5318. doi: 10.1038/ncomms6318.

Paudel, P., McDonald, F.J., and Fronius, M. (2021). The delta subunit of epithelial sodium channel in humans-a potential player in vascular physiology. *Am J Physiol Heart Circ Physiol* 320(2)**,** H487-H493. doi: 10.1152/ajpheart.00800.2020.

Pergel, E., Veres, I., Csigi, G.I., and Czirjak, G. (2021). Translocation of TMEM175 Lysosomal Potassium Channel to the Plasma Membrane by Dynasore Compounds. *Int J Mol Sci* 22(19). doi: 10.3390/ijms221910515.

Phillips, C.M., Johnson, A.M., Stamatovic, S.M., Keep, R.F., and Andjelkovic, A.V. (2023). 20 kDa isoform of connexin-43 augments spatial reorganization of the brain endothelial junctional complex and lesion leakage in cerebral cavernous malformation type-3. *Neurobiol Dis* 186**,** 106277. doi: 10.1016/j.nbd.2023.106277.

Picollo, A., and Pusch, M. (2005). Chloride/proton antiporter activity of mammalian CLC proteins ClC-4 and ClC-5. *Nature* 436(7049)**,** 420-423. doi: 10.1038/nature03720.

Quinodoz, M., Rutz, S., Peter, V., Garavelli, L., Innes, A.M., Lehmann, E.F., et al. (2025). De novo variants in LRRC8C resulting in constitutive channel activation cause a human multisystem disorder. *EMBO J* 44(2)**,** 413-436. doi: 10.1038/s44318-024-00322-y.

Raut, S.K., Singh, K., Sanghvi, S., Loyo-Celis, V., Varghese, L., Singh, E.R., et al. (2024). Chloride ions in health and disease. *Biosci Rep* 44(5). doi: 10.1042/BSR20240029.

Rocca, C., Soda, T., De Francesco, E.M., Fiorillo, M., Moccia, F., Viglietto, G., et al. (2023). Mitochondrial dysfunction at the crossroad of cardiovascular diseases and cancer. *J Transl Med* 21(1)**,** 635. doi: 10.1186/s12967-023-04498-5.

Ruan, Z., Osei-Owusu, J., Du, J., Qiu, Z., and Lu, W. (2020). Structures and pH-sensing mechanism of the proton-activated chloride channel. *Nature* 588(7837)**,** 350-354. doi: 10.1038/s41586-020-2875-7.

Salmeri, M., Motta, C., Anfuso, C.D., Amodeo, A., Scalia, M., Toscano, M.A., et al. (2013). VEGF receptor-1 involvement in pericyte loss induced by Escherichia coli in an in vitro model of blood brain barrier. *Cell Microbiol* 15(8)**,** 1367-1384. doi: 10.1111/cmi.12121.

Sancak, Y., Markhard, A.L., Kitami, T., Kovacs-Bogdan, E., Kamer, K.J., Udeshi, N.D., et al. (2013). EMRE is an essential component of the mitochondrial calcium uniporter complex. *Science* 342(6164)**,** 1379-1382. doi: 10.1126/science.1242993.

Sandoval, H., Ibanez, B., Contreras, M., Troncoso, F., Castro, F.O., Caamano, D., et al. (2025). Extracellular Vesicles From Preeclampsia Disrupt the Blood-Brain Barrier by Reducing CLDN5. *Arterioscler Thromb Vasc Biol* 45(2)**,** 298-311. doi: 10.1161/ATVBAHA.124.321077.

Sanghvi, S.K., Gabrilovich, D., Raut, S.K., Gopalan, A., Singh, A., Bhachu, H.R., et al. (2025). Biophysical characterization of anion channels in mitochondrion-endoplasmic-reticulum contact sites. *Biophys J* 124(10)**,** 1599-1608. doi: 10.1016/j.bpj.2025.04.002.

Schreiber, R., Ousingsawat, J., Wanitchakool, P., Sirianant, L., Benedetto, R., Reiss, K., et al. (2018). Regulation of TMEM16A/ANO1 and TMEM16F/ANO6 ion currents and phospholipid scrambling by Ca(2+) and plasma membrane lipid. *J Physiol* 596(2)**,** 217-229. doi: 10.1113/JP275175.

Schreurs, M.P., Houston, E.M., May, V., and Cipolla, M.J. (2012). The adaptation of the blood-brain barrier to vascular endothelial growth factor and placental growth factor during pregnancy. *FASEB J* 26(1)**,** 355-362. doi: 10.1096/fj.11-191916.

Shen, J., Xu, G., Zhu, R., Yuan, J., Ishii, Y., Hamashima, T., et al. (2019). PDGFR-beta restores blood-brain barrier functions in a mouse model of focal cerebral ischemia. *J Cereb Blood Flow Metab* 39(8)**,** 1501-1515. doi: 10.1177/0271678X18769515.

Shin, H.W., and Takatsu, H. (2019). Substrates of P4-ATPases: beyond aminophospholipids (phosphatidylserine and phosphatidylethanolamine). *FASEB J* 33(3)**,** 3087-3096. doi: 10.1096/fj.201801873R.

Sternak, M., Bar, A., Adamski, M.G., Mohaissen, T., Marczyk, B., Kieronska, A., et al. (2018). The Deletion of Endothelial Sodium Channel alpha (alphaENaC) Impairs Endothelium-Dependent Vasodilation and Endothelial Barrier Integrity in Endotoxemia in Vivo. *Front Pharmacol* 9**,** 178. doi: 10.3389/fphar.2018.00178.

Sun, Y., Chen, X., Zhang, X., Shen, X., Wang, M., Wang, X., et al. (2017). Corrigendum: beta2-Adrenergic Receptor-Mediated HIF-1alpha Upregulation Mediates Blood Brain Barrier Damage in Acute Cerebral Ischemia. *Front Mol Neurosci* 10**,** 392. doi: 10.3389/fnmol.2017.00392.

Tejada, M.A., Stople, K., Hammami Bomholtz, S., Meinild, A.K., Poulsen, A.N., and Klaerke, D.A. (2014). Cell volume changes regulate slick (Slo2.1), but not slack (Slo2.2) K+ channels. *PLoS One* 9(10)**,** e110833. doi: 10.1371/journal.pone.0110833.

Thirugnanam, K., Gupta, A., Nunez, F., Prabhudesai, S., Pan, A.Y., Nauli, S.M., et al. (2023). Brain microvascular endothelial cells possess a second cilium that arises from the daughter centriole. *Front Mol Biosci* 10**,** 1250016. doi: 10.3389/fmolb.2023.1250016.

Tian, Y., Wang, Y., Zhao, Y., Liu, C., Zhang, X., Zhang, Y., et al. (2025). LRRC8A in endothelial cells contributes to the aberrant blood-brain barrier integrity in ischaemic stroke. *Stroke Vasc Neurol*. doi: 10.1136/svn-2024-003675.

Tipparaju, S.M., Barski, O.A., Srivastava, S., and Bhatnagar, A. (2008). Catalytic mechanism and substrate specificity of the beta-subunit of the voltage-gated potassium channel. *Biochemistry* 47(34)**,** 8840-8854. doi: 10.1021/bi800301b.

Tu, H., Nelson, O., Bezprozvanny, A., Wang, Z., Lee, S.F., Hao, Y.H., et al. (2006). Presenilins form ER Ca2+ leak channels, a function disrupted by familial Alzheimer's disease-linked mutations. *Cell* 126(5)**,** 981-993. doi: 10.1016/j.cell.2006.06.059.

van Veen, S., Martin, S., Van den Haute, C., Benoy, V., Lyons, J., Vanhoutte, R., et al. (2020). ATP13A2 deficiency disrupts lysosomal polyamine export. *Nature* 578(7795)**,** 419-424. doi: 10.1038/s41586-020-1968-7.

Varela, L., Hendry, A.C., Cassar, J., Martin-Escolano, R., Cantoni, D., Ossa, F., et al. (2022). A Zn2+-triggered two-step mechanism of CLIC1 membrane insertion and activation into chloride channels. *J Cell Sci* 135(15). doi: 10.1242/jcs.259704.

Venturi, E., Sitsapesan, R., Yamazaki, D., and Takeshima, H. (2013). TRIC channels supporting efficient Ca(2+) release from intracellular stores. *Pflugers Arch* 465(2)**,** 187-195. doi: 10.1007/s00424-012-1197-5.

Wang, H., Xu, M., Kong, Q., Sun, P., Yan, F., Tian, W., et al. (2017). Research and progress on ClC‑2 (Review). *Mol Med Rep* 16(1)**,** 11-22. doi: 10.3892/mmr.2017.6600.

Wang, Q.C., Zheng, Q., Tan, H., Zhang, B., Li, X., Yang, Y., et al. (2016). TMCO1 Is an ER Ca(2+) Load-Activated Ca(2+) Channel. *Cell* 165(6)**,** 1454-1466. doi: 10.1016/j.cell.2016.04.051.

Wu, L.Y., Song, Y.J., Zhang, C.L., and Liu, J. (2023). K(V) Channel-Interacting Proteins in the Neurological and Cardiovascular Systems: An Updated Review. *Cells* 12(14). doi: 10.3390/cells12141894.

Xiang, X., Feng, Z., Wang, L., Wang, D., Li, T., Yang, J., et al. (2025). CLIC1 and IFITM2 expression in brain tissue correlates with cognitive impairment via immune dysregulation in sepsis and Alzheimer's disease. *Int Immunopharmacol* 155**,** 114628. doi: 10.1016/j.intimp.2025.114628.

Xu, Y., Yang, Y., Yang, J., Cui, J., Yan, J., Jiang, J., et al. (2024). Glycine Receptor Beta Subunit (GlyR-beta) Promotes Potential Angiogenesis and Neurological Regeneration during Early-Stage Recovery after Cerebral Ischemia Stroke/Reperfusion in Mice. *J Integr Neurosci* 23(8)**,** 145. doi: 10.31083/j.jin2308145.

Yang, X., Meyer, K., and Friedl, A. (2013). STAT5 and prolactin participate in a positive autocrine feedback loop that promotes angiogenesis. *J Biol Chem* 288(29)**,** 21184-21196. doi: 10.1074/jbc.M113.481119.

Yoast, R.E., Emrich, S.M., Zhang, X., Xin, P., Johnson, M.T., Fike, A.J., et al. (2020). The native ORAI channel trio underlies the diversity of Ca(2+) signaling events. *Nat Commun* 11(1)**,** 2444. doi: 10.1038/s41467-020-16232-6.

Zhang, C., Tong, F., Zhou, B., He, M., Liu, S., Zhou, X., et al. (2024a). TMC6 functions as a GPCR-like receptor to sense noxious heat via Galphaq signaling. *Cell Discov* 10(1)**,** 66. doi: 10.1038/s41421-024-00678-9.

Zhang, J., Yuan, H.K., Chen, S., and Zhang, Z.R. (2022). Detrimental or beneficial: Role of endothelial ENaC in vascular function. *J Cell Physiol* 237(1)**,** 29-48. doi: 10.1002/jcp.30505.

Zhang, R.J., Yin, Y.F., Xie, X.J., and Gu, H.F. (2020). Acid-sensing ion channels: Linking extracellular acidification with atherosclerosis. *Clin Chim Acta* 502**,** 183-190. doi: 10.1016/j.cca.2019.12.027.

Zhang, X., Shao, J., Wang, C., Liu, C., Hao, H., Li, X., et al. (2024b). TMC7 functions as a suppressor of Piezo2 in primary sensory neurons blunting peripheral mechanotransduction. *Cell Rep* 43(4)**,** 114014. doi: 10.1016/j.celrep.2024.114014.

Zhang, X., Yang, Q., Zhang, R., Zhang, Y., Zeng, W., Yu, Q., et al. (2024c). Sodium Danshensu ameliorates cerebral ischemia/reperfusion injury by inhibiting CLIC4/NLRP3 inflammasome-mediated endothelial cell pyroptosis. *Biofactors* 50(1)**,** 74-88. doi: 10.1002/biof.1991.

Zhang, Y., Ding, X., Miao, C., and Chen, J. (2019). Propofol attenuated TNF-alpha-modulated occludin expression by inhibiting Hif-1alpha/ VEGF/ VEGFR-2/ ERK signaling pathway in hCMEC/D3 cells. *BMC Anesthesiol* 19(1)**,** 127. doi: 10.1186/s12871-019-0788-5.

Zheng, W., Rawson, S., Shen, Z., Tamilselvan, E., Smith, H.E., Halford, J., et al. (2023). TMEM63 proteins function as monomeric high-threshold mechanosensitive ion channels. *Neuron* 111(20)**,** 3195-3210 e3197. doi: 10.1016/j.neuron.2023.07.006.

Zhu, D., Su, Y., Fu, B., and Xu, H. (2018). Magnesium Reduces Blood-Brain Barrier Permeability and Regulates Amyloid-beta Transcytosis. *Mol Neurobiol* 55(9)**,** 7118-7131. doi: 10.1007/s12035-018-0896-0.

Zong, P., Feng, J., Li, C.X., Jellison, E.R., Yue, Z., Miller, B., et al. (2024). Activation of endothelial TRPM2 exacerbates blood-brain barrier degradation in ischemic stroke. *Cardiovasc Res* 120(2)**,** 188-202. doi: 10.1093/cvr/cvad126.

Zuccolo, E., Kheder, D.A., Lim, D., Perna, A., Nezza, F.D., Botta, L., et al. (2019a). Glutamate triggers intracellular Ca(2+) oscillations and nitric oxide release by inducing NAADP- and InsP3 -dependent Ca(2+) release in mouse brain endothelial cells. *J Cell Physiol* 234(4)**,** 3538-3554. doi: 10.1002/jcp.26953.

Zuccolo, E., Laforenza, U., Negri, S., Botta, L., Berra-Romani, R., Faris, P., et al. (2019b). Muscarinic M5 receptors trigger acetylcholine-induced Ca(2+) signals and nitric oxide release in human brain microvascular endothelial cells. *J Cell Physiol* 234(4)**,** 4540-4562. doi: 10.1002/jcp.27234.

Zuccolo, E., Lim, D., Kheder, D.A., Perna, A., Catarsi, P., Botta, L., et al. (2017). Acetylcholine induces intracellular Ca2+ oscillations and nitric oxide release in mouse brain endothelial cells. *Cell Calcium* 66**,** 33-47. doi: 10.1016/j.ceca.2017.06.003.
